# Understanding the Dynamics of Sulfur Droplets Formation in Lean‐Electrolyte and Low‐Temperature Lithium–Sulfur Batteries

**DOI:** 10.1002/advs.202410628

**Published:** 2024-12-06

**Authors:** Qi Qi, Fangyi Shi, Jingya Yu, Yiyuan Ma, Feiyang Chen, Wei Lv, Wing‐Cheung Law, Shu Ping Lau, Zheng‐Long Xu

**Affiliations:** ^1^ Department of Industrial and Systems Engineering The Hong Kong Polytechnic University Hung Hom Hong Kong 999077 P. R. China; ^2^ Department of Applied Physics The Hong Kong Polytechnic University Hung Hom Hong Kong 999077 P. R. China; ^3^ State Key Laboratory of Ultraprecision Machining Technology Department of Industrial and Systems Engineering The Hong Kong Polytechnic University Hung Hom Hong Kong 999077 P. R. China; ^4^ Shenzhen Geim Graphene Center Engineering Laboratory for Functionalized Carbon Materials Tsinghua Shenzhen International Graduate School Tsinghua University Shenzhen 518055 P. R. China; ^5^ Research Institute for Smart Energy Research Institute for Advanced Manufacturing The Hong Kong Polytechnic University Hung Hom Hong Kong 999077 P. R. China; ^6^ Hong Kong Polytechnic University Shenzhen Research Institute Shenzhen Guangdong 518057 P. R. China

**Keywords:** in‐situ observations, liquid sulfur, lithium–sulfur batteries, reaction kinetics

## Abstract

Lithium–sulfur batteries (LSBs) afford great promises as the next‐generation rechargeable batteries due to the high energy density and low cost of sulfur cathodes. Lean‐electrolyte condition constitutes the prerequisite for high‐energy LSBs, but the insulating sulfur particles hinder capacity utilization, especially at low temperatures. Here, the electrochemical generation of liquid sulfur droplets in the LSB system are studied and elucidate the polysulfide oxidation reaction (SOR) kinetics under different electrolyte/sulfur (E/S) ratios and low‐temperature conditions. The real‐time observations under in situ optical and Raman microscopies indicate that the formation of liquid sulfur during SOR is independent of the E/S ratio and can be preserved over a wide range of operating temperatures. Quantification of the polysulfide reactant concentrations and the amounts of the liquid sulfur product under different charging conditions reveal pseudo‐zero‐order kinetics and E/S ratio‐dependent reaction constants for the SOR process. In addition, under extreme conditions of −20 °C and E/S ratio of 5 µL mg^−1^, liquid sulfur can still be preserved by following the rapid SOR kinetics. These findings provide new insights into the liquid sulfur generation dynamics in Li─S chemistry, which enables a deeper understanding of the effects of the E/S ratio and working temperature on the oxidation kinetics in LSBs.

## Graphical abstract

Liquid sulfur formation on CNFs substrate reveals pseudo‐zero‐order kinetics and E/S ratio‐dependent reaction rates for the SOR process. Even in lean electrolytes and under low temperatures at −20 °C, liquid sulfur can still be preserved due to the rapid reaction kinetics.

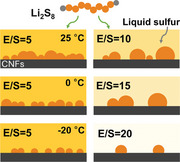

## 1 Introduction

Sulfur is an attractive cathode material in electrochemical energy storage systems for high‐energy metal‐sulfur batteries, such as lithium sulfur batteries (LSBs), sodium sulfur batteries (SSBs), and magnesium‐sulfur batteries (MSBs) configurations.^[^ ^1^ ^]^ Different from the intercalation‐type cathodes like LiFePO_4_ and LiCoO_2_ in lithium‐ion batteries (LIBs), sulfur cathodes undergo reversible conversion reactions with metal sulfides like Li_2_S, thus delivering an exceptionally high capacity of 1672 mAh g^−1^ and energy density of 2600 Wh kg^−1^ in the case of LSBs.^[^ ^2^, ^3^, ^4^ ^]^ While considerable efforts have been dedicated to improving the practical performance of LSBs by regulating the sulfur reduction reaction during discharging, the sulfide oxidation reaction (SOR) kinetics during charging, which are equally critical, have received much less attention.^[^ ^5^ ^]^ The prevailing understanding posits that lithium sulfides are oxidized through a solid‐liquid‐solid process involving the formation of liquid polysulfides and solid *β*‐phase sulfur in an LSB.^[^ ^6^ ^]^ However, this paradigm was recently challenged by the observation of supercool liquid sulfur forming on the surface of the Ni grid through electrochemical cycling.^[^ ^7^ ^]^ The same phenomenon can also be captured on other substrates, such as palladium, platinum, indium tin oxide, transition metal dichalcogenides (TMD), and graphene.^[^ ^8^, ^9^ ^]^ In the subsequent studies, the nucleation and growth behavior of liquid sulfur were investigated through operando observations.^[^ ^10^ ^]^ By leveraging the rapid reaction kinetics of liquid sulfur on a 3D nickel‐based host, they achieved remarkable high‐rate performance LSBs.^[^ ^11^ ^]^ The flowable and reshapable liquid sulfur at room temperature opens new opportunities toward approaching high‐power and high‐energy LSBs. Nevertheless, the conditions for forming this liquid phase and its role in modifying the SOR kinetics remain unclear.

Various efforts have been devoted to exploring the formation of liquid sulfur at room temperature in electrochemical systems.^[^ ^12^ ^]^ In an optical microscopy‐equipped micro‐cell, supercooled liquid sulfur droplets were initially observed from polysulfide oxidation reaction on a nickel metal grid. The formation of liquid sulfur at 155 °C below its melting temperature provides new insights into the SOR process in LSBs.^[^ ^7^ ^]^ Unfortunately, it was subsequently revealed that the liquid sulfur is thermodynamically metastable. On the surface of TMD flakes (i.e., MoS_2_), liquid sulfur droplets proliferated on the basal plane, whereas solid sulfur precipitated from the edge to the basal plane, eventually crystallizing and enveloping the basal plane of MoS_2_ with insulating sulfur crystals, thereby stalling the SOR reaction.^[^ ^8^ ^]^ Similar phenomena were observed in 2D materials, like graphite, WS_2_, and WSe_2_. To preserve the appealing liquid phase sulfur, we designed a sulfur‐vacant MoS_2‐x_ substrate by annealing MoS_2_ flakes in the H_2_ atmosphere at high temperatures,^[^ ^13^ ^]^ which could maintain the liquid sulfur throughout the entire charging process, even at low temperatures down to −40 °C. To further elucidate the liquid‐to‐liquid SOR process, we have comprehensively investigated the sulfur growing dynamics on the surface of graphene^[^ ^9^ ^]^ which illustrated the less dependence of charging capacities for liquid sulfur on current densities than solid sulfur, thereby providing an effective strategy for fast‐charging LSBs.

Despite these advancements, the studies utilized catholytes as sulfur resources with excessive electrolyte volumes (i.e., electrolyte‐to‐sulfur E/S ratios > 20 µL mg^−1^). It has been calculated that the practical energy densities of LSBs would be unable to rival those of commercial LIB if the E/S ratios surpass 7.5 µL mg^−1^.^[^ ^14^ ^]^ In addition, the cathode kinetic challenges are exacerbated under lean electrolyte conditions, leading to reduced capacity utilization and limited cycle life.^[^ ^15^ ^]^ To date, research on liquid sulfur SOR has been in its infancy. Several critical questions remain open. For example, is the generation of liquid sulfur sustainable under low E/S ratios? What is the correlation between E/S ratios and the growing kinetics of liquid sulfur? How can liquid sulfur chemistry effectively be utilized in advanced LSBs under lean‐electrolyte and low‐temperature conditions?

In this work, we employ an operando optical micro‐cell to observe and quantify the SOR process across a spectrum of E/S ratios and temperatures, aiming to fully understand their reaction pathways and kinetic parameters. Carbon nanofibers (CNF) are selected as the substrate to eliminate the impacts of catalytic effect or unpredictable interactions of polar materials to polysulfides on the kinetic factors. By visualizing the evolutions of sulfur morphology and Raman spectra, we demonstrate that the formation of liquid sulfur is not contingent upon the E/S ratios and operating temperatures, with liquid sulfur even manifesting at E/S ratio = 5 µL mg^−1^ and −20 °C. By applying classical nucleation theory to quantify sulfur droplet size and reactant concentrations, we reveal the pseudo‐zero‐order reaction kinetics characteristic of polysulfide redox processes. Contrary to expectations, the reaction constant, indicative of the sulfur growth rate, escalates with decreasing E/S ratios, is due to the augmented accessibility of polysulfides to the active CNF substrate surface. During charging at reduced operational temperatures (from 25 to −20 °C), the SOR persists in a pseudo‐zero‐order reaction mode, albeit with attenuated reaction rates. This work provides compelling experimental evidence of SOR kinetics and mechanistic insights that extend the knowledge of Li‐S redox chemistry under harsh conditions of lean electrolytes and low temperatures.

## 2 Result and Discussion

### 2.1 Sulfur Droplet Formation Kinetics Under Different E/S Ratios

**Figure 1:**
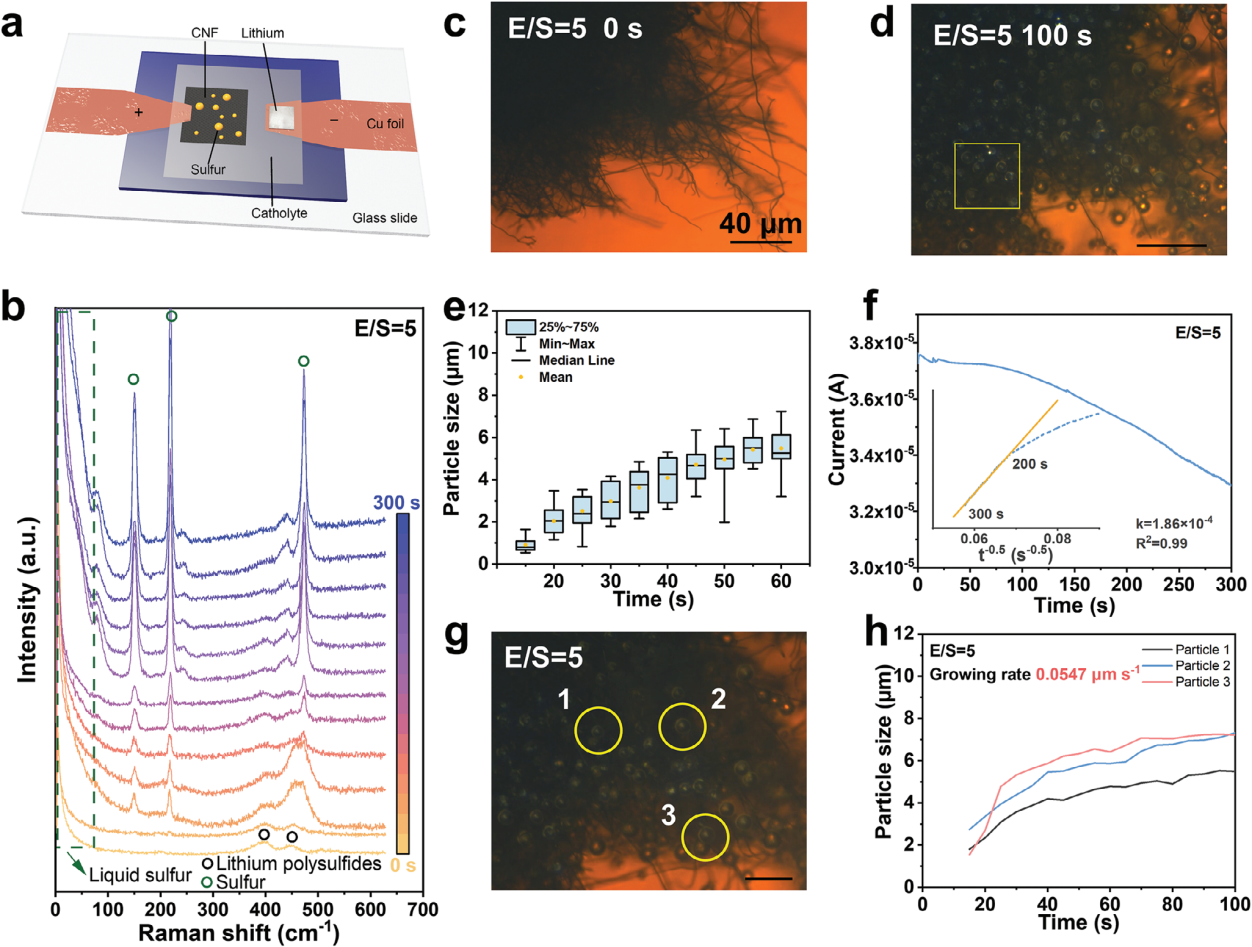
a) Scheme of optical micro‐cell. b) Raman test of the micro‐cell with an E/S ratio of 5 µL mg^−1^ at a constant voltage of 4 V. Raman curves were collected every 25 s during the 300 s’ charging period. c,d) Optical images were taken at initial and 100 s with an E/S ratio of 5 µL mg^−1^. A square is marked with yellow for droplet distribution calculation. e) Particle size analysis of the sample with an E/S ratio of 5 µL mg^−1^. f) The reaction current for the sample with an E/S ratio of 5 µL mg^−1^ (insert figure is the linear fitting of the current to t^−0.5^). g) Three droplets marked as 1, 2, and 3 for the calculation of liquid sulfur growing rate in (h).

An optical microcell was designed to observe the sulfur generation behaviors under different E/S ratios, as shown in **Figure** **1**a. In detail, the micro‐cell contains a piece of thin CNF film as a cathode current collector, a lithium metal pasted on Cu foil as anode, and different concentrations of Li_2_S_8_ in 1,3‐dioxolane/1,2‐dimethoxyethane (DOL/DME) solution as the catholyte. The E/S ratio was adjusted to 5, 10, 15, and 20 µL mg^−1^ using 0.78, 0.4, 0.26, and 0.2 mol L^−1^ Li_2_S_8_, respectively. These micro‐cells were sealed between the Si/SiO_2_ substrate and transparent glass covers for optical and Raman observations. The open‐circuit voltage of the micro‐cell is ≈2.3 V (Figure S1, Supporting Information), close to that of real LSB using Li_2_S_8_ catholyte cathode. Applying an external voltage to the micro‐cell triggers the SOR process (Li_2_S_8_ – 2e – 2Li^+^ → S_8_). An overview shows sulfur droplets in all the micro‐cells with different E/S ratios considered in this work, and the sulfur grows more aggressively under low E/S ratio conditions (Figure S2, Movies S1–S4, Supporting Information). These preliminary findings indicate a natural tendency for liquid sulfur generation in LSBs and highlight the significant influence of E/S ratios on the SOR kinetics.

To illustrate the phase evolution during the SOR process, in situ Raman characterization was performed on an electrochemical cell with an E/S ratio of 5 µL mg^−1^. Figure 1b shows two broad peaks at 400 and 448 cm^−1^ for fresh cells corresponding to long‐chain polysulfides. After charging, three new peaks appeared at 153, 220, and 473 cm^−1^, corresponding to the asymmetric, symmetric S─S bending, and S─S stretching in S_8_ rings.^[^ ^6^ ^]^ A distinctive Rayleigh wing in the external vibration range (10–100 cm^−1^) was also observed, attributable to the long‐range disorder of S_8_ molecules within the droplets. Similar Raman peaks were also observed for the electrochemical cells with E/S ratios of 10, 15, and 20 µL mg^−1^ (Figure S3, Supporting Information). It is noted that the sulfur droplets may convert into crystalline phases under laser beam exposure, as inferred by the Raman peaks at 28, 44, 51, 63, and 88 cm^−1^ for *α*‐phase S_8_ in Figure S3c (Supporting Information). The morphologies of solid sulfur particles were detected under SEM, which shows uniform sulfur particles among CNF with average particle sizes of 6 µm (Figure S4, Supporting Information), consistent with optical observations.

The sulfur growing dynamics at different E/S ratios were then evaluated by plotting the average droplet sizes at 5 s intervals within a defined area of 40 µm × 40 µm (see the yellow frames in Figures 1c,d; Figures S5a–c, Supporting Information). For instance, in the electrochemical cell with an E/S ratio = 5 µL mg^−1^, the quantified droplet sizes in Figure 1e and Table S1 (Supporting Information) show a gradual increase from 0.907 µm at 15 s to 5.487 µm at 60 s, yielding an average growth rate of 0.102 µm s^−1^. The mean deviation increases from 0.285 to 0.795 µm and the standard deviation from 37.4 to 97.6% over the same interval. This trend suggests ongoing nucleation and coalescence of sulfur droplets during charging, characterized by the continuous emergence of small nuclei amidst the growth of other droplets on the CNF substrate (Figure S6, Supporting Information). Similar phenomena were observed for the electrochemical microcells with E/S ratios = 10, 15, and 20 µL mg^−1^ (Figures S5d–f, Supporting Information). Comparing the average growing rate, deviation of size distributions and the final sizes of sulfur droplets formed under different E/S ratios reveals several interesting insights: i) the low E/S ratio can benefit the sulfur growing rate (i.e., 0.102 µm s^−1^ for E/S ratio = 5 µL mg^−1^, 0.0615 µm s^−1^ for E/S ratio = 20 µL mg^−1^), potentially due to increased polysulfide accessibility to the CNF current collector, which supports droplet growth in lean‐electrolyte conditions; ii) sulfur droplets growing at an E/S ratio of 5 µL mg^−1^ exhibited smaller standard deviations throughout the charging process, suggesting more constrained nucleation of new droplets at higher E/S ratios. These concurrent processes dominate the reaction at different stages, as reflected in the reaction currents and the growth kinetics of the sulfur droplets.^[^ ^16^ ^]^

To further delineate the sulfur growing kinetics, we dissected the charge transfer‐controlled and diffusion‐limited processes separately. Under the constant voltage, the SOR process initiated the charge transfer‐controlled nucleation process, followed by a diffusion‐limited growing process. According to the Cottrell equation, the polysulfide diffusion coefficient can be calculated by fitting the reaction current (A) versus t^−0.5^ (second^−0.5^) (see the details in Note S1, Supporting Information).^[^ ^17^ ^]^ Figure 1f shows the current‐time curve of the sample with an E/S ratio of 5 µL mg^−1^ alongside the fitting result for current versus t^−0.5^. It indicates a slope of the charge transfer‐controlled process before 200 s. Building on this analysis, we focused on three individual droplets (labeled 1, 2, and 3) that exhibited direct growth from the SOR process, rather than through coalescence with neighboring droplets, as depicted in Figure 1g. The temporal evolution of their sizes is documented in Table S2 (Supporting Information). Figure 1h compiles the size‐time trajectories for these droplets, reflecting the charge transfer process with an average growth rate of 0.0547 µm s^−1^. The observed decline in growth rate over time is consistent with reaction‐limited kinetics, indicative of the depletion of active polysulfides.

Then, we scrutinized the droplet growing rate by analyzing three representative droplets marked as 1, 2, and 3 in micro‐cells with E/S ratios of 10, 15, and 20 µL mg^−1^ (Figures S7a–c, Supporting Information). Their reaction kinetics were interrogated using the Cottrell equation (Figure S8, Supporting Information), and their sizes were charted against the charging duration of up to 100 s. In the sample with an E/S ratio of 10 µL mg^−1^, the reaction was predominantly governed by charge transfer kinetics before 120 s with a sulfur‐droplet growing rate of 0.0692 µm s^−1^, which surpasses the 0.0547 µm s^−1^ for E/S ratio = 5 µL mg^−1^. This discrepancy is ascribed to the pronounced coalescence process competing with the independent growth of sulfur droplets at the lower E/S ratio. In the samples with E/S ratios of 15 and 20 µL mg^−1^, the droplet size‐time slops can also be divided into two segments according to the fitting results in Figures S8e,f (Supporting Information). Comparing the sulfur growth rates during the charge transfer‐controlled process across E/S ratios from 5 to 20 µL mg^−1^ reveals a narrow range of variation (from 0.05 to 0.07 µm s^−1^, with a deviation within 0.02 µm s^−1^). Conversely, during the diffusion‐controlled process, the growth rates are markedly slower (0.019 and 0.0149 µm s^−1^ for samples with E/S ratios of 15 and 20 µL mg^−1^, respectively), signifying the rate‐determining step. The diffusion coefficients (*D*) for polysulfides at different E/S ratios were calculated and tabulated in Table S3 (Supporting Information). Notably, for the E/S ratio of 5 µL mg^−1^, *D* is calculated to be 1.22 × 10^−8^ cm^2^ s^−1^, which is lower than those for E/S ratios of 10 and 15 µL mg^−1^, potentially due to increased viscosity.

To gain a deeper understanding of SOR kinetics, we determined the reaction order and the reaction rate constants using the following relationship:

(1) $$k\;{\left[Li_{2}S_{8}\right]}^{n}=-\frac{d[Li_{2}S_{8}]}{dt}$$

where *k* represents the reaction constant for the oxidation of Li_2_S_8_ per active site, *n* represents the reaction order, [Li_2_S_8_] is the concentration of Li_2_S_8_, which, in this work, is represented by the projected area of sulfur droplets [A] from optical imaging.^[^ ^18^ ^]^ Consequently, Equation (1) can be reformulated as:

(2) $$k_{s}\;A^{n}=-\frac{dA}{dt}$$

**Figure 2:**
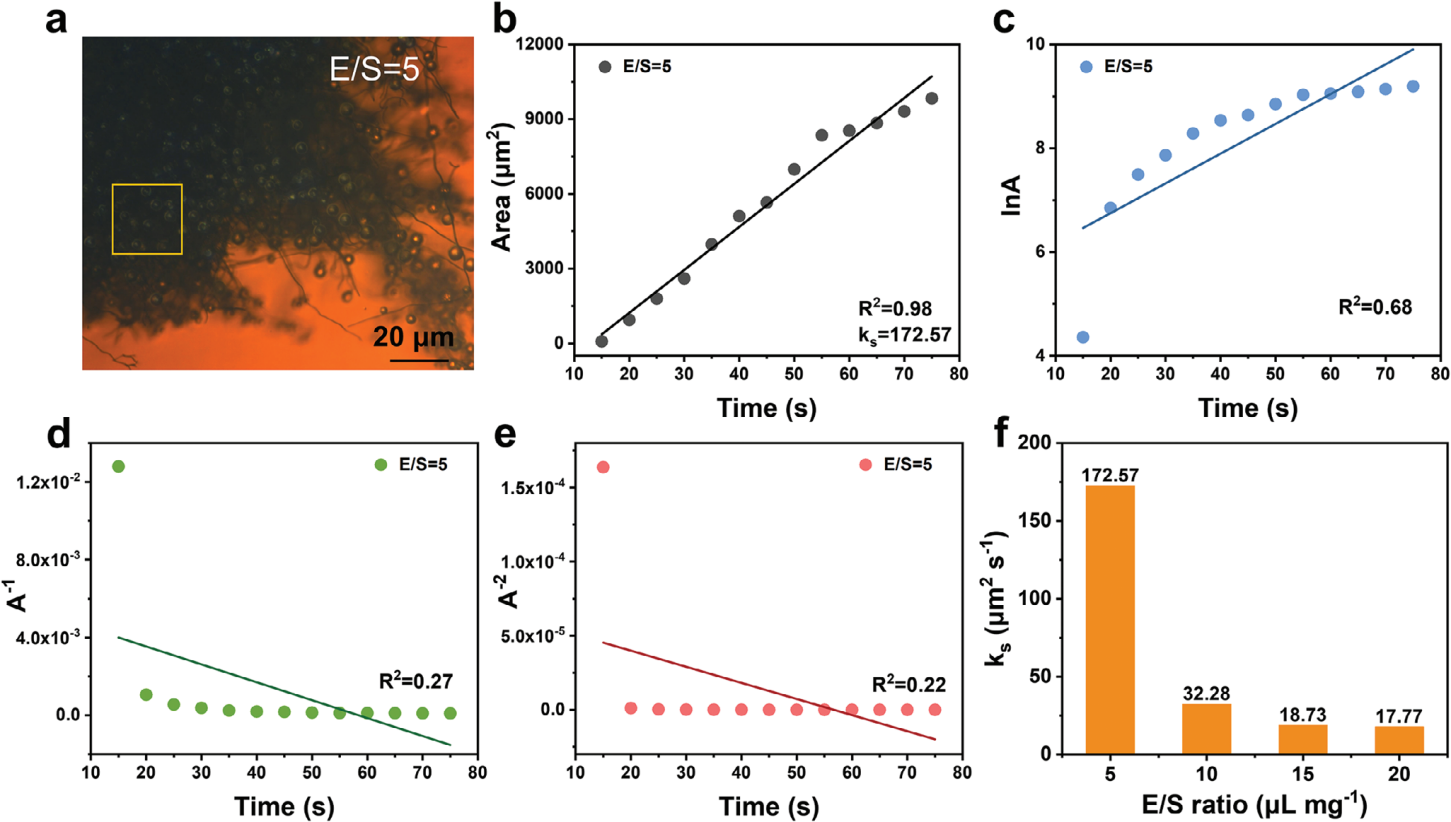
a) Optical image of the sample with an E/S ratio of 5 µL mg^−1^ and a 40 µm × 40 µm selection for reaction order calculation marked with a yellow frame. Linear fitting curves of liquid sulfur area change with time: b) plot of the area with time, c) plot of the logarithm of the area with time, d) plot of the reciprocal of the area with time, e) plot of the negative quadratic of the area with time. f) Comparison of the k_s_ values among different E/S ratios.

The reaction order, *n*, can be obtained by fitting the experimental data [A] to various rate expressions with typical models. For example, the zero, first, second, and third‐order kinetics refer to the linear relationship between [A], ln[A], [A]^−1^, [A]^−2^ with time (*t*). For the electrochemical microcell with an E/S ratio = 5 µL mg^−1^ (**Figures** **2**a–e), the coefficient of determination, R^2^, values are 0.98, 0.68, 0.27, 0.22 for zero, first, second, and third‐order models, respectively, thus indicating pseudo‐zero‐order reaction kinetics for the oxidation of polysulfides to liquid sulfur.^[^ ^19^ ^]^ The fitting results for micro‐cells with E/S ratios of 10, 15, and 20 µL mg^−1^ (Figure S9, Supporting Information) are consistent with those for an E/S ratio of 5 µL mg^−1^, suggesting an E/S ratio‐independent reaction order for liquid sulfur formation. According to Equation (2), the rate constants (*k_s_*) for zero‐order reactions are mathematically equal to the growing rate of sulfur area, which were calculated to be 172.57, 32.28, 18.73, and 17.77 µm^2^ s^−1^ for the micro‐cells with E/S ratios of 5, 10, 15 and 20 µL mg^−1^, respectively (Figure 2f). Notably, such pseudo‐zero‐order kinetics have been extensively investigated in reactions such as cobalt‐single atom catalyzed sulfur reduction and platinum‐catalyzed decomposition of NO_2_, where reaction rates were determined by the available surface area for (electro)chemical reactions.^[^ ^20^, ^21^ ^]^ In this work, the surface areas of the CNF substrate were assumed to be constant. The high *k_s_* at lower E/S ratios can be attributed to the increased utilization degree of CNF surface that facilitates the nucleation and growth of flowable liquid sulfur droplets. This quantitative analysis suggests the most rapid reaction kinetics for the sample with an E/S ratio of 5 µL mg^−1^, diverging from the expected or observed behavior for solid sulfur electrochemistry. The accelerated kinetics for sulfur droplet formation are ascribed to its charge‐transfer controlled reaction kinetics and the enhanced accessibility of polysulfides on the CNF substrate.

**Figure 3:**
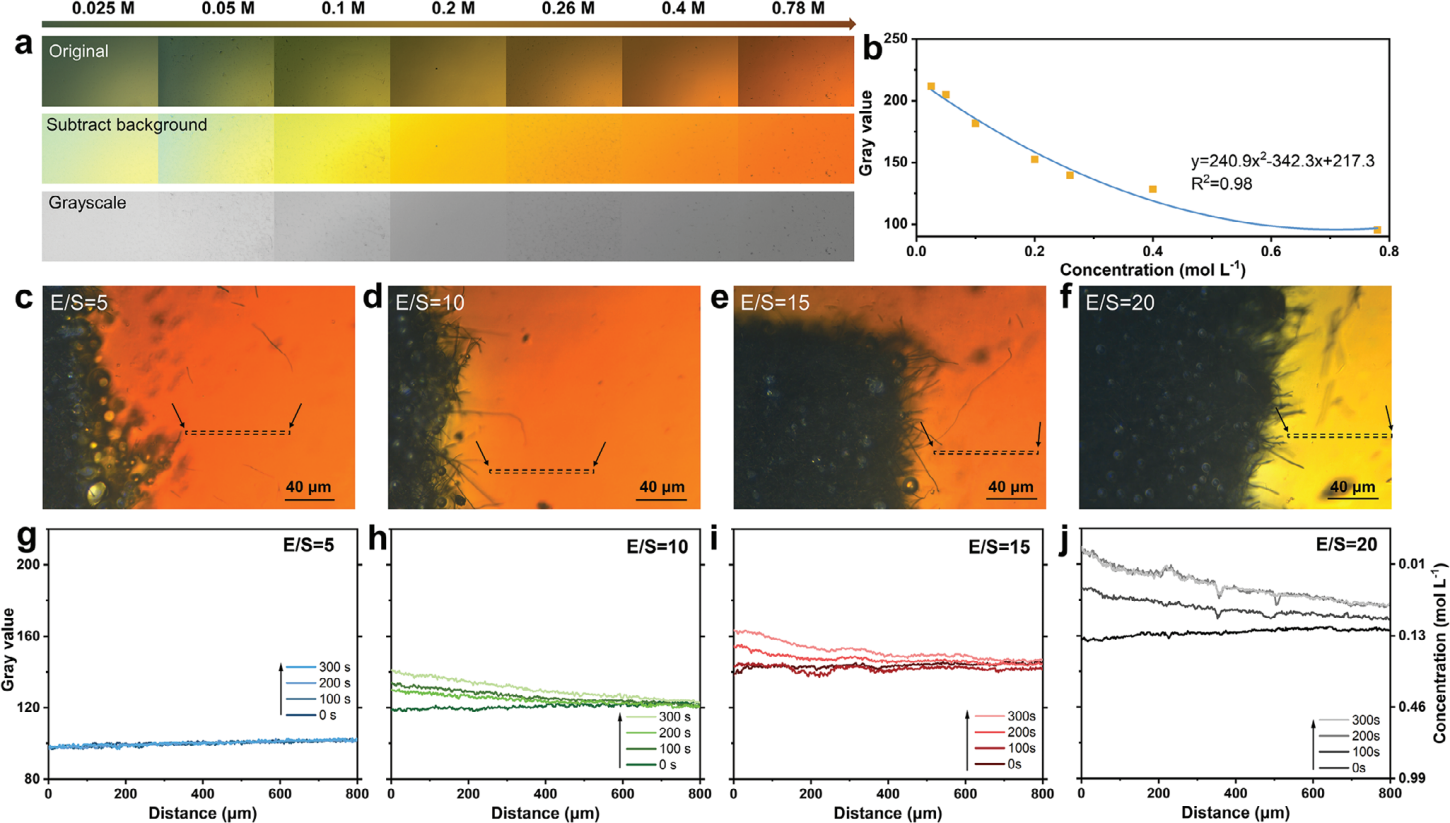
a) Optical RGB images and the corresponding grayscale images of Li_2_S_8_ catholyte with increasing concentrations (0.025, 0.05, 0.1, 0.2, 0.26, 0.4 and 0.78 m). b) Quadratic fitting curve between gray value and Li_2_S_8_ concentration. c–f) Optical images of samples with E/S ratios of 5, 10, 15, and 20 µL mg^−1^. An 800 µm stripe was marked with a dash frame in each sample for polysulfide diffusion study. g–j) Gray value (left y‐axis) and Li_2_S_8_ concentration (right y‐axis) change as the distance from the CNF substrate to the bulky catholyte during different reaction depths: initial, 100, 200, and 300 s with E/S ratios of 5, 10, 15, and 20 µL mg^−1^.

The above discussions underscore the critical role of polysulfide diffusion kinetics in the SOR process, which has been widely overlooked or inaccessible in previous studies. To quantify the diffusion and consumption of polysulfides, we capitalized on the distinctive color change observable in our transparent micro‐cells. By establishing a correlation between polysulfide concentration and the catholyte's color, it becomes feasible to calibrate the colors of catholyte solutions against known polysulfide concentrations. Specifically, we prepared a series of Li_2_S_8_ solutions (0.025–0.78 mol L^−1^ corresponding to E/S ratios from 160 to 5 µL mg^−1^) and measured the corresponding gray intensities in the RGB gray images (**Figure** **3**a). Following mathematical modeling, a quadratic relationship between the gray value and Li_2_S_8_ concentration was established (Figure 3b and Note S2, Supporting Information), similar to the method developed for tissue recognition in the biology field.^[^ ^22^ ^]^ The evolution of the catholyte concentration along the polysulfide diffusion path can reflect the diffusion‐limited SOR processes.^[^ ^23^ ^]^ As shown in Figure S10b (Supporting Information), the color derivation from the CNF substrate to the bulk catholyte at 300 s can be identified. Moreover, the depth of charging (or consumption of polysulfides) can be estimated by monitoring the color change proximal to the CNF substrate within the microcells.

Having established the methodology to quantify polysulfide concentrations, we first chose a spot‐shaped area to calculate the catholyte concentration evolution during charging (see the white square in Figure S2, Supporting Information). It shows that the gray intensities increase significantly from 0 to 300 s for E/S ratios of 10, 15, and 20 µL mg^−1^, suggesting the conspicuous consumption of Li_2_S_8_ for sulfur droplets growing (Figure S11, Supporting Information). Interestingly, no apparent change of the gray intensities was detected for E/S ratio = 5 µL mg^−1^ during the whole SOR reaction period, which can be ascribed to the low‐capacity utilization or/and rapid polysulfides diffusion in the concentrated polysulfide solution. The spatial evolutions of polysulfide concentration during SOR were then studied by plotting 800 µm‐length stripes from the CNF substrate to bulky catholyte (see the dash frames in Figures 3c–f, marked by arrows) from the initial state to 300 s. The calculated polysulfide concentrations at 0, 100, 200, and 300 s is plotted as a function of the measurement length in Figures 3g–j. We also listed the polysulfide concentrations at the start and ending points of the stripes after full charging (at 300 s) in Table S4 (Supporting Information). At the outset (0 s), all samples exhibit constant gray values along the 800 µm length, indicating a uniform distribution of polysulfides before the SOR process. No polysulfide concentration gradient was observed over the 800 µm strip for an E/S ratio of 5 µL mg^−1^ throughout the charging process (Figure 3g).

In contrast, Figures 3h,i reveal grayscale gradients for E/S ratios of 10 and 15 µL mg^−1^ at 300 s, displaying decreasing gray values from 0 µm (CNF substrate) to 800 µm (bulk catholyte), indicative of significant polysulfide consumption at the CNF/catholyte interface and insufficient polysulfide diffusion for SOR (suggesting diffusion‐limited kinetics). The polysulfide concentration gradients over the 800 µm‐length strips at 300 s were calculated to be ≈30% for E/S ratios of 10 and 15 µL mg^−1^ cell, aligning with their comparable sulfur growth rates. For the sample with a flooded electrolyte (E/S ratio of 20 µL mg^−1^ in Figure 3j), a pronounced polysulfide concentration gradient was observed after 100 s, implying a more substantial influence of polysulfide diffusion on the SOR process. From 200 to 300 s, the polysulfide concentration near the CNF substrate decreased by 87.3%, due to significant polysulfide utilization and sluggish diffusion from the bulk catholyte to the CNF, which may lead to cell polarization and suboptimal sulfur utilization in practical batteries. These quantitative analyses conclude that the ROS process involves liquid sulfur droplet formation with pseudo‐zero‐order reaction kinetics, which are independent of polysulfide concentrations (E/S ratios). However, the SOR kinetics are highly contingent upon polysulfide diffusion. Counterintuitively, a concentrated catholyte with low E/S ratios can enhance polysulfide diffusion and conversion rates, offering advantages for high‐power lithium‐liquid sulfur cells.

### 2.2 Sulfur Generation Kinetics at Low Temperatures

**Figure 4:**
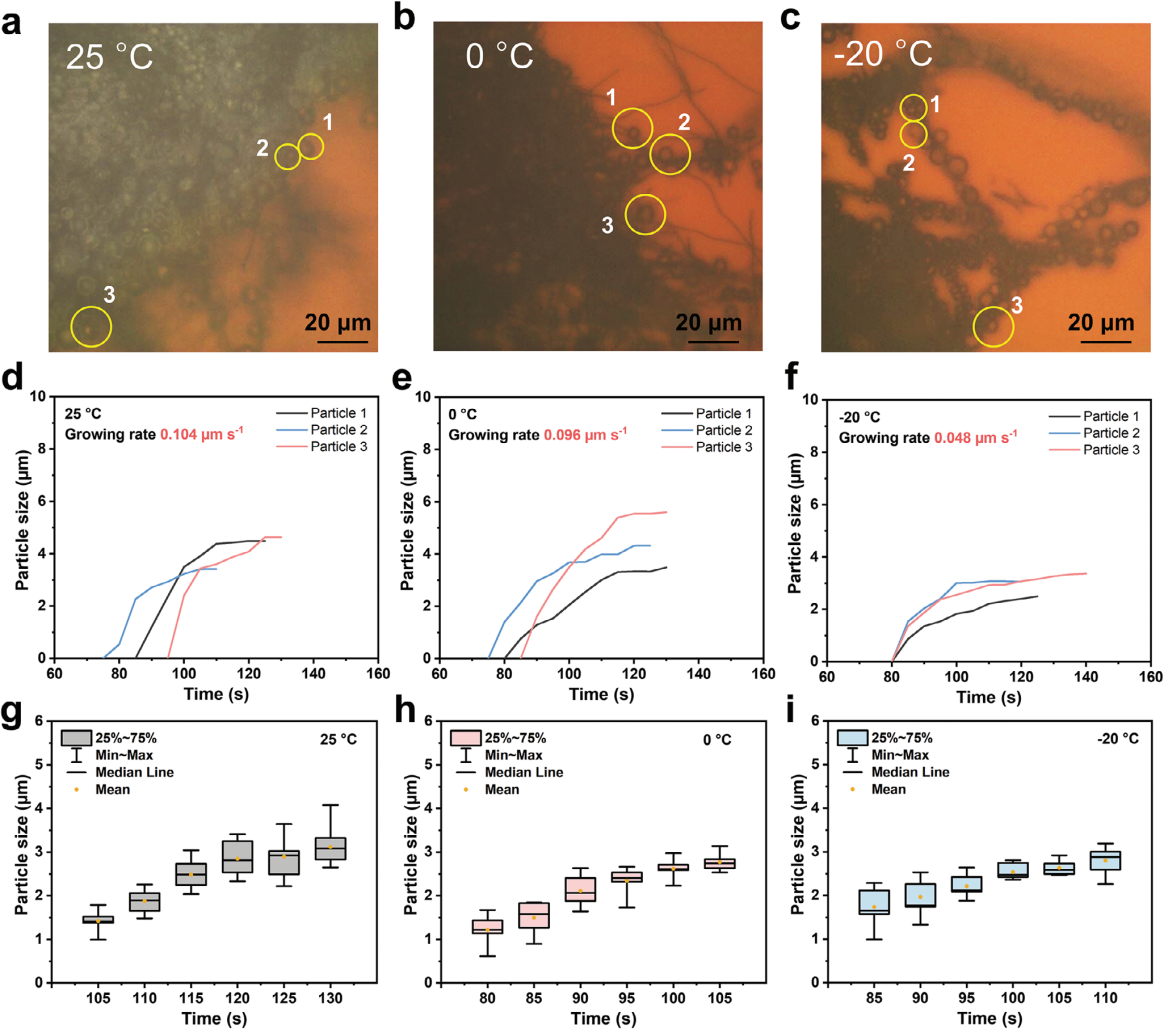
Three droplets were chosen for each sample with an E/S ratio of 5 µL mg^−1^ in (a) room temperature, b) 0 °C, and c) −20 °C. The corresponding liquid sulfur growing rates in (d) room temperature, e) 0 °C, and (f) −20 °C. Liquid sulfur size distribution in (g) room temperature, h) 0 °C, and i) −20 °C.

Low temperature has been considered to be another detrimental factor for the reaction kinetics in LSBs, particularly under low E/S ratio conditions. To probe the sulfur generation behaviors at low temperatures, we utilized a temperature‐controlled platform to adjust the operating temperature from 25 to −20 °C with an E/S ratio = 5 µL mg^−1^.^[^ ^13^ ^]^ Sequential images captured from Movies S5–S7 (Supporting Information) (**Figures** **4**a–c) illustrate the formation of sulfur droplets on CNFs as corroborated by Raman spectroscopy (Figure S12, Supporting Information) at 25, 0, and −20 °C, respectively. Initial SOR current responses, modeled by the Cottrell equation (Figure S13, Supporting Information), indicate a transition from charge‐transfer‐limited and diffusion‐controlled kinetics at 0 °C to exclusively charge transfer‐limited kinetics at −20 °C, suggesting a significant deceleration of the SOR process at lower temperatures. This postulation is further substantiated by the observed average sulfur growth rates of 0.104, 0.096, and 0.048 µm s^−1^ for representative droplets at temperatures ranging from 25 to −20 °C (Figures 4d–f). The droplet sizes are ≈3 µm (Figures 4g–i). When we further decrease the temperature to −40 °C, no liquid sulfur appears on CNF, as shown in Figure S14 (Supporting Information), possibly due to the sluggish SOR kinetics.

To ascertain the reaction order and reaction constant of the SOR process at low temperatures, we selected discernible CNF fibers of 30 µm in length to measure sulfur droplet size distributions at various temperatures (Figures S15a–c, Supporting Information). The average areas of sulfur droplets, plotted against reaction time, exhibit a clear linear relationship, indicative of pseudo‐zero‐order reaction kinetics at low temperatures (Figures S15d–f, Supporting Information). The rate constants (*k_s_*) were calculated to be 44.57, 30.54, and 25.47 µm^2^ s^−1^ at 25, 0, and −20 °C, respectively. In conjunction with the preceding discussions, it is reasonable to conclude that SOR kinetics are markedly attenuated at low temperatures, attributable to the intrinsic conversion kinetics rather than polysulfide diffusion in lean electrolyte conditions. Notably, the *k_s_* values are relatively lower than those at room temperature, which may result from the selection of single fiber samples for our calculations due to the limited resolution of sulfur droplets at low‐temperature conditions. In summary, liquid sulfur can still be generated under low‐temperature (−20 °C) and lean‐electrolyte (E/S ratio = 5 µL mg^−1^) conditions, with formation kinetics influenced by the slowed conversion dynamics.

### 2.3 Application of Liquid Sulfur in Lean‐Electrolyte LSBs

**Figure 5:**
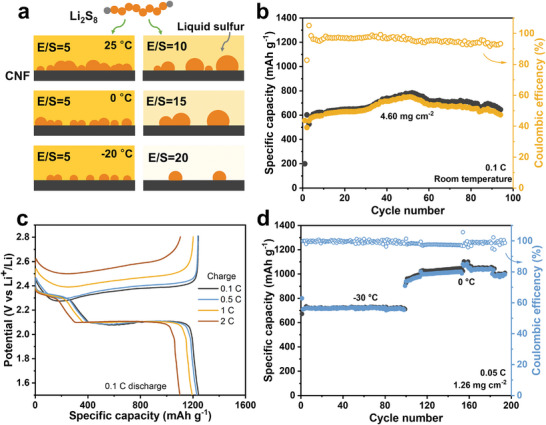
a) Scheme of liquid sulfur generation behavior under different temperatures and E/S ratios. b) Cyclic performance of CNF/catholyte cathodes with a sulfur loading of 4.60 mg cm^−2^. c) Rate capacities of CNF/catholyte cathodes with an E/S ratio of 5 µL mg^−1^. The discharging rate is 0.1C, and the charging rates are 0.1, 0.5, 1, and 2C. d) Cycling performance under low temperatures of −30 and 0 °C at 0.05C.

Our comprehensive investigations on SOR kinetics under lean‐electrolyte and low‐temperature conditions in electrochemical micro‐cells are summarized in **Figure** **5**a. It recommends the formation of liquid sulfur droplets with favorable reaction kinetics across various E/S ratios and operating temperatures. We assembled Li─S coin cells with CNF/catholyte cathodes to assess the practical application of sulfur droplets. To identify the liquid sulfur formation, we used a homemade coin cell with a transparent silica window for in situ Raman characterization (Figure S16a, Supporting Information). During the LSV test from 2.1 to 2.8 V at a scan rate of 1 mV s^−1^ (Figure S16b, Supporting Information), a distinct polysulfide oxidation peak is observed at ≈2.6 V. Under optical microscopy (Figure S16c, Supporting Information), we can visualize the bright droplets surrounding CNFs, which are identified as liquid sulfur with a Rayleigh wing in the external vibration region (<100 cm^−1^) (Figure S16d, Supporting Information). Thus, liquid sulfur can be produced as the SOR product in practical Li─S cells.

Finally, coin cells with a high sulfur loading of 4.6 mg cm^−2^ and a low E/S ratio of 7 µL mg^−1^ was cycled at 0.1 C at room temperature (1C = 1672 mA g^−1^) (Figure 5b). Without any ameliorations to the electrolyte compositions, interlayers, or catalytic materials, our Li─S cells involving the formation of liquid sulfur could reach remarkable capacities of ≈555 mAh g^−1^ at the 1st cycle followed by a gradual capacity increase to 751 mAh g^−1^ after 50 cycles, which can be attributed to the activation process caused by the redistribution of active materials in the 3D carbon host in high sulfur loading cells. The cells maintained a capacity of 645 mAh g^−1^ at the 92nd cycle, leading to a high‐capacity retention of 86%. For fast‐charging measurements, the Li─S cells were discharged at 0.1C and charged at increasing current densities from 0.1 to 2C. Figure 5c shows a high capacity of 1105 mAh g^−1^ at a high charging rate of 2C, corresponding to a theoretical capacity utilization of 66%, which is a challenging feat in solid‐sulfur‐based LSBs with similar sulfur/carbon cathodes.^[^ ^24^, ^25^ ^]^ Besides, we cycled the coin cells under low temperatures of 0 and −30 °C in Figure 5d. Under −30 °C, the Li─S cell shows an initial capacity of 720 mAh g^−1^ and excellent capacity retention over 100 cycles at 0.05C with an extremely low‐capacity decay rate of 0.014% per cycle. Increasing the operating temperature to 0 °C resulted in a capacity boost to 926 mAh g^−1^, which stabilized at 1009 mAh g^−1^ after 100 cycles. High sulfur utilization at low temperatures and lean electrolytes, typically unattainable with conventional sulfur/carbon cathodes, can be ascribed to this study's unique liquid sulfur growth dynamics.

## 3 Conclusion

In this work, for the first time, we disclosed the formation and preservation of sulfur droplets under lean electrolyte and low‐temperature conductions through operando visualization and Raman characterizations, thus expanding our understanding of the SOR kinetics and informing strategies for achieving high‐power and high‐energy LSBs. By quantifying the current responses and droplet sizes, we find that the SOR process follows a pseudo‐zero‐order reaction kinetic mode and an E/S ratio‐dependent reaction rate, where the low E/S ratio of 5 µL mg^−1^ is beneficial for expediting conversion reactions. This counterintuitive discovery is ascribed to the influence of polysulfide diffusion kinetics under both flooded electrolyte and low‐temperature conditions, which have been largely overlooked in conventional solid sulfur/lithium electrochemical systems. By correlating the color of polysulfide catholytes with their concentrations, we successfully quantified the diffusion process of polysulfides across various stages of the SOR and under different operational conditions. This approach has yielded unprecedented evidence that elucidates whether the SOR kinetics are governed by diffusion or reaction processes. These profound insights resolve the fundamental reaction kinetics in SOR and offer valuable suggestions for electrode design in advanced LSBs. For example, the cathode with competitive sulfur loadings can reach high sulfur utilization at low temperatures (720 mAh g^−1^ after 100 cycles at −30 °C) and lean electrolyte (1105 mAh g^−1^ at 2C for E/S ratio = 7 µL mg^−1^) conditions. Leveraging the unique properties of liquid sulfur provides an unexplored pathway toward optimizing LSBs under challenging conditions.

## Conflict of Interest

The authors declare no conflict of interest.

## Author Contributions

Q.Q. and F.S. contributed equally to this work. Z.L.X. conceived the idea and supervised the project, Q.Q., F.S. and Z.L.X. designed and conducted the experiments, F.S., J.Y., Y.M., F.C., W.L., W.C.L., and S.P.L. support the characterization of materials and devices fabrication, Q.Q., F.S., and Z.L.X. wrote the manuscript, all the authors commented on the manuscript.

## Supporting information



Supporting Information

Supplemental Movie 1

Supplemental Movie 2

Supplemental Movie 3

Supplemental Movie 4

Supplemental Movie 5

Supplemental Movie 6

Supplemental Movie 7

## Data Availability

The data that support the findings of this study are available from the corresponding author upon reasonable request.
