# Supplementary material for: Understanding the Dynamics of Sulfur Droplets Formation in Lean‐Electrolyte and Low‐Temperature Lithium–Sulfur Batteries
Source: Adv Sci (Weinh). 2024 Dec 6;12(4):2410628. doi: 10.1002/advs.202410628 (PMC11775558; doi:10.1002/advs.202410628)
Supplement: Supplementary file 1 — Supporting Information [file ADVS-12-2410628-s005.docx]

**Supporting Information**

**Understanding the dynamics of sulfur droplets formation in lean-electrolyte and low-temperature lithium sulfur batteries**

Qi Qi^1#^, Fangyi Shi^2#^, Jingya Yu^1,3^, Yiyuan Ma^1,3^, Feiyang Chen^1^, Wei Lv^4^, Wing-Cheung Law^1^, Shu Ping Lau^2^*, Zheng-Long Xu^1,3,5,6^*

*1. Department of Industrial and Systems Engineering, The Hong Kong Polytechnic University, Hung Hom, Hong Kong, 999077, P.R. China.*

*2. Department of Applied Physics, The Hong Kong Polytechnic University, Hung Hom, Hong Kong, 999077, P.R. China.*

*3. State Key Laboratory of Ultraprecision Machining Technology, Department of Industrial and Systems Engineering, The Hong Kong Polytechnic University, Hung Hom, Hong Kong, 999077, P.R. China.*

*4. Shenzhen Geim Graphene Center, Engineering Laboratory for Functionalized Carbon Materials, Tsinghua Shenzhen International Graduate School, Tsinghua University, Shenzhen, 518055, China.*

*5. Research Institute for Smart Energy, Research Institute for Advanced Manufacturing, The Hong Kong Polytechnic University, Hung Hom,* Hong Kong, *999077,* P.R. China.

*6. Hong Kong Polytechnic University Shenzhen Research Institute, Shenzhen 518057, Guangdong, P.R. China.*

Corresponding authors. Email: [zhenglong.xu@polyu.edu.hk](mailto:zhenglong.xu@polyu.edu.hk), apsplau@polyu.edu.hk

# These authors contributed equally to this work.

**Experimental methods**

**Preparation of CNFs:** CNF was prepared through an electrospinning method.^[1]^ Polyacrylonitrile (PAN) was purchased from Sigma, which was dissolved in 20 mL dimethylformamide (DMF). The as prepared mixture was used as the precursor for electrospinning and magnetically stirred for 24 hours at room temperature. The solution was then electro-spun at 14.0 kV. The distance from the needle to the current collector (Al foil) was around 10 cm and the flow rate was 0.5 mL h^-1^. The product PAN film was thermalized at 220 ºC for 3 h in the air and then to 1050 ºC in Argon atmosphere to obtain carbon nanofiber (CNF). The ramp rate is 3 ºC per minute.

**Catholyte preparation:** Li_2_S_8_ catholyte with a series of concentration including 0.2 M, 0.26 M, 0.4 M and 0.78 M was prepared by mixing sulfur and Li_2_S and dissolving them in DOL/DME (1:1) electrolyte. The amounts of sulfur are 1.4 M, 1.82 M, 2.8 M and 5.46 M, respectively, and the amounts of Li_2_S are 0.2 M, 0.25 M, 0.4 M and 0.78 M accordingly. 0.5 M LiTFSI and 0.1 M LiNO_3_ were added to each catholyte as additive.

**Optical cell fabrication:** Silicon wafer was cut into a square of 1 cm × 1 cm and taped in the middle of a glass slide. CNF was prepared in a suitable size as cathode current collector on wafer, which was shown in **Figure 1a**. Lithium metal was used as anode, which together with CNF were both connected to the external circuit through copper foil. A suitable-sized cover glass was placed above them and sealed by Kapton tape with two voids near the anode. The catholyte prepared above was injected into the optical cell through the void by capillary and sealed by vacuum grease in the glove box filled with Ar atmosphere.

**In situ optical microscopy:** The optical cell was charged with a constant voltage of 4 V using an electrochemical workstation (CHI 760E) and observed under optical microscope (Leica Dm 2700M). A charging voltage of 3 V was also carried out for comparison as **Figure S17** shows. Liquid sulfur droplets were pointed out by yellow arrows, but the number of them were too small to analyze. Images and videos were recorded through the camera connected to the optical microscope. The images were taken with 50× microscope objective and the videos were recorded at 30 frames per second for 300 s per sample. All the images were analyzed through Image J.

**In situ Raman spectroscopy:** The confirmation of liquid or solid sulfur was carried out through confocal Raman microscopy (WITec Inc.) with laser of 532 nm. The images and videos were recorded at 1 frame per second by 50× microscope objective. The accumulations are 2 and the integration time is 5 s. The Raman curves in **Figure 1b** and **Figure S3** were collected every 25 seconds during charging.

**Low-temperature testing:** The optical cell was prepared to be tested by a temperature controlled machine (DSC600). The temperature of the test stage was adjusted by Linksys32 software and an LNP95 liquid nitrogen pump. During the test, dry nitrogen kept working to cool down the test stage and avoid the water in the air condensing on the observation window. Despite this, the observation clarity was partially impaired. The micro-cell was charged at 4 V, 5 V and 6 V under room temperature, 0 ºC and -20 ºC, respectively. The devices were prepared in smaller size to fit the facility’s observation stage.

**Materials characterization and electrochemical measurements:** The morphology and mapping images of CNF was taken by field emission electron microscopy (Tescan MIRA) with X-ray analysis of elements.

The CR2032 coin cell were assembled in an Ar-filled glovebox. 1 cm × 1 cm CNF (the mass of it is around 1.5 mg and the thickness is around 90 μm) was prepared as sulfur host and 0.78 M Li_2_S_8_ was added into it (E/S ratio is 5 μL mg^-1^). The amount of Li_2_S_8_ catholyte was 5-20 μL and the loading of sulfur is 1-4.6 mg. Then a piece of 2400 Celgard separator (19 mm) was put on the above of CNF. Following this, 10 μL blank electrolyte was added to the separator with a piece of lithium metal (the diameter is 15.6 mm, the thickness if 45 μm) on it. A rGO/S cathode was used in the coin cell tested under low temperatures. The coin cells were all tested in a voltage of 1.5 V to 2.8 V. Accordingly, the E/S ratio of the coin cell in Figure b-d is 7, 10 and 15 μL mg^-1^ in total.


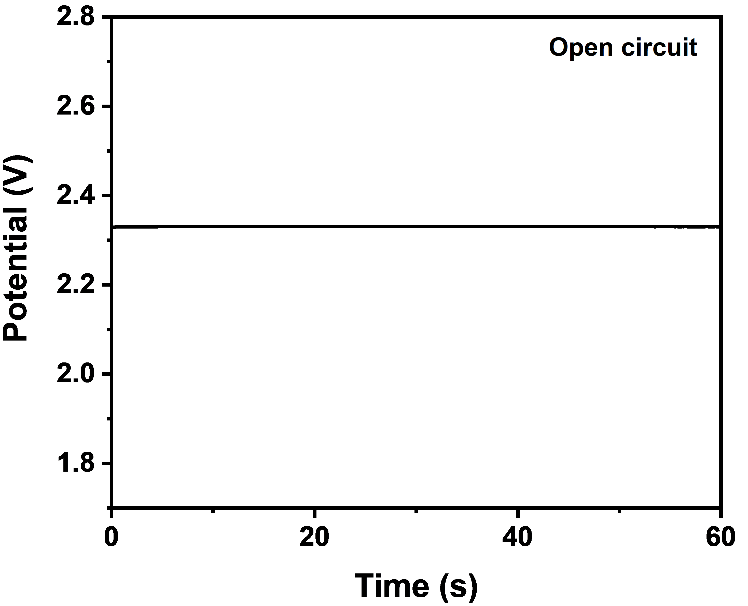


**Figure S1** Open circuit of the optical micro-cell.


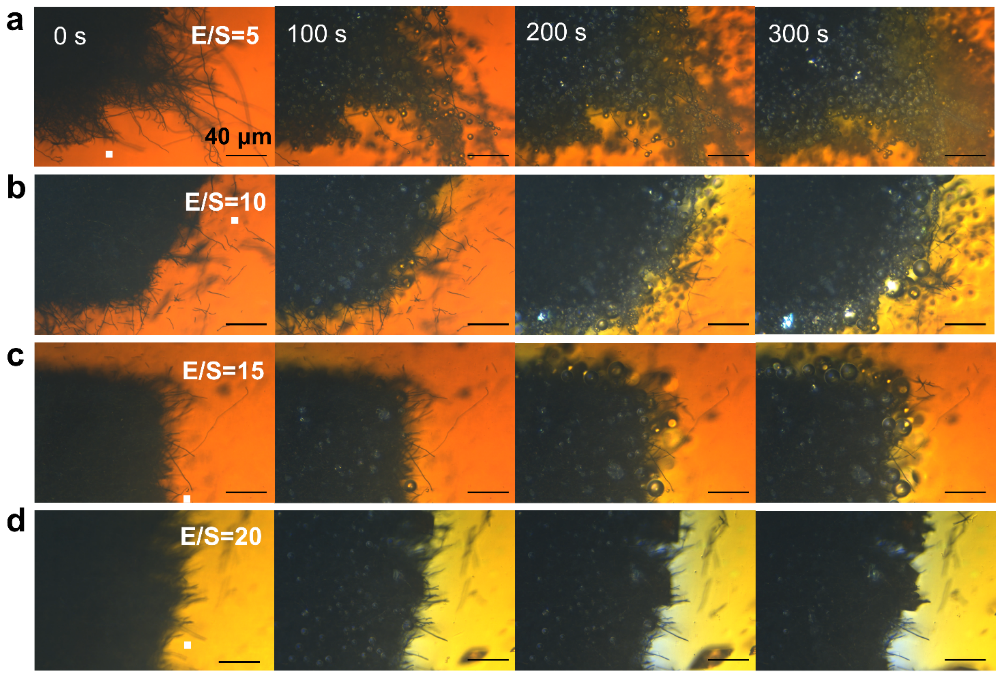


**Figure S2** (a-d) Optical images of micro-cells with various E/S ratios of 5, 10, 15 and 20 μL mg^-1^. The white dots in the first column are chosen for concentration analysis in Figure 2b. All the samples were tested under a constant voltage of 4 V.


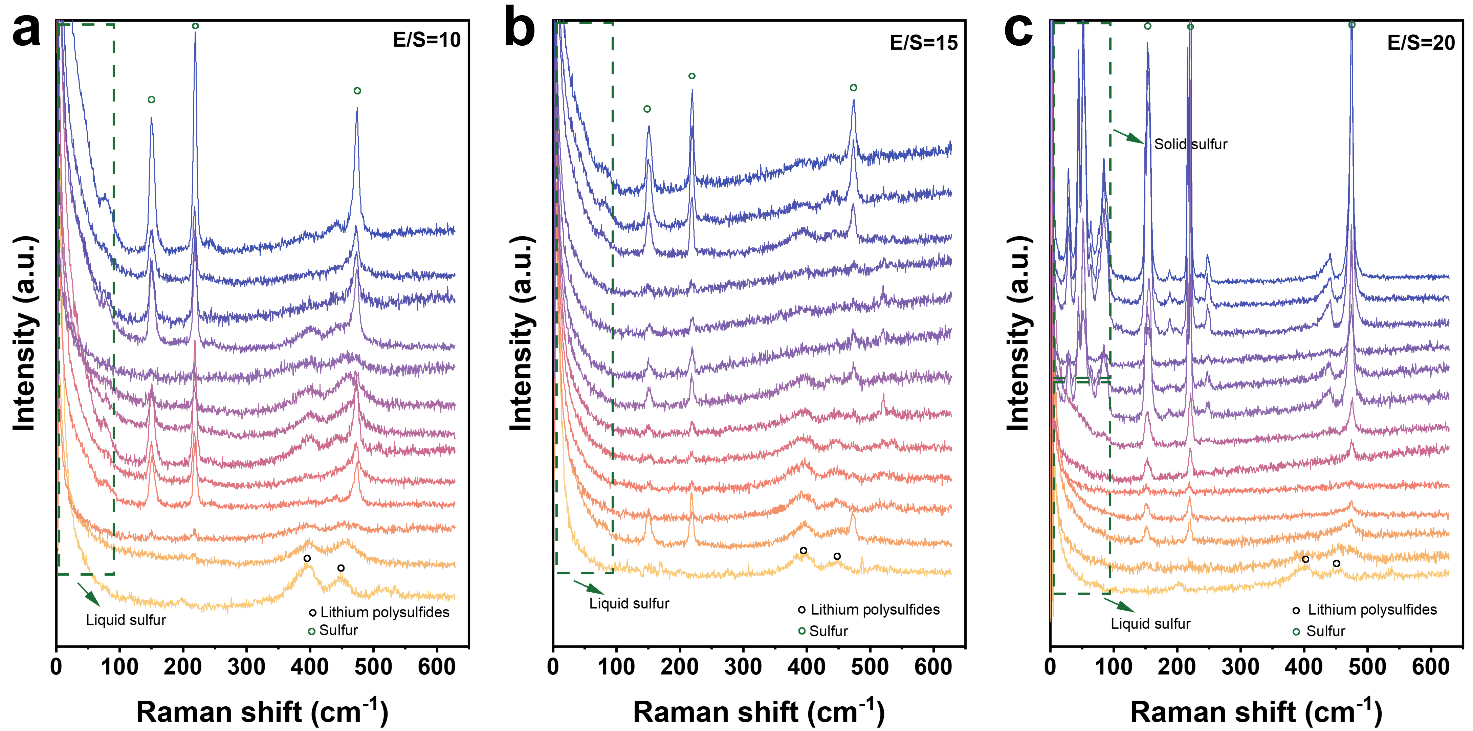


**Figure S3** (a-c) Raman tests of micro-cells with different E/S ratio of 10, 15 and 20 μL mg^-1^. The low frequency spectrum below 100 cm^-1^ is marked with green dash frames.


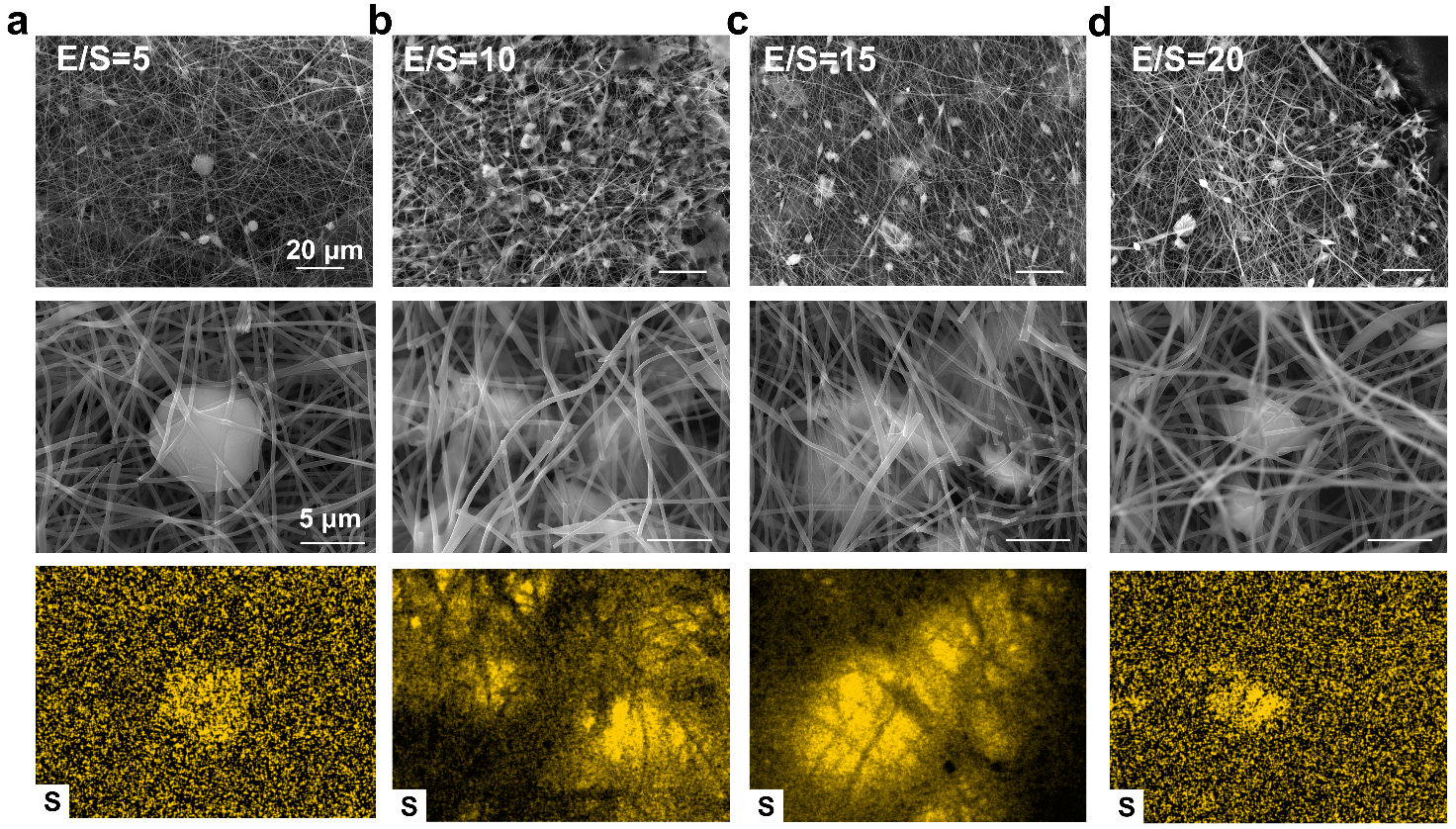


**Figure S4** (a-d) SEM images and the corresponding mapping images of CNF from micro-cells with different E/S ratio of 5, 10, 15 and 20 μL mg^-1^.


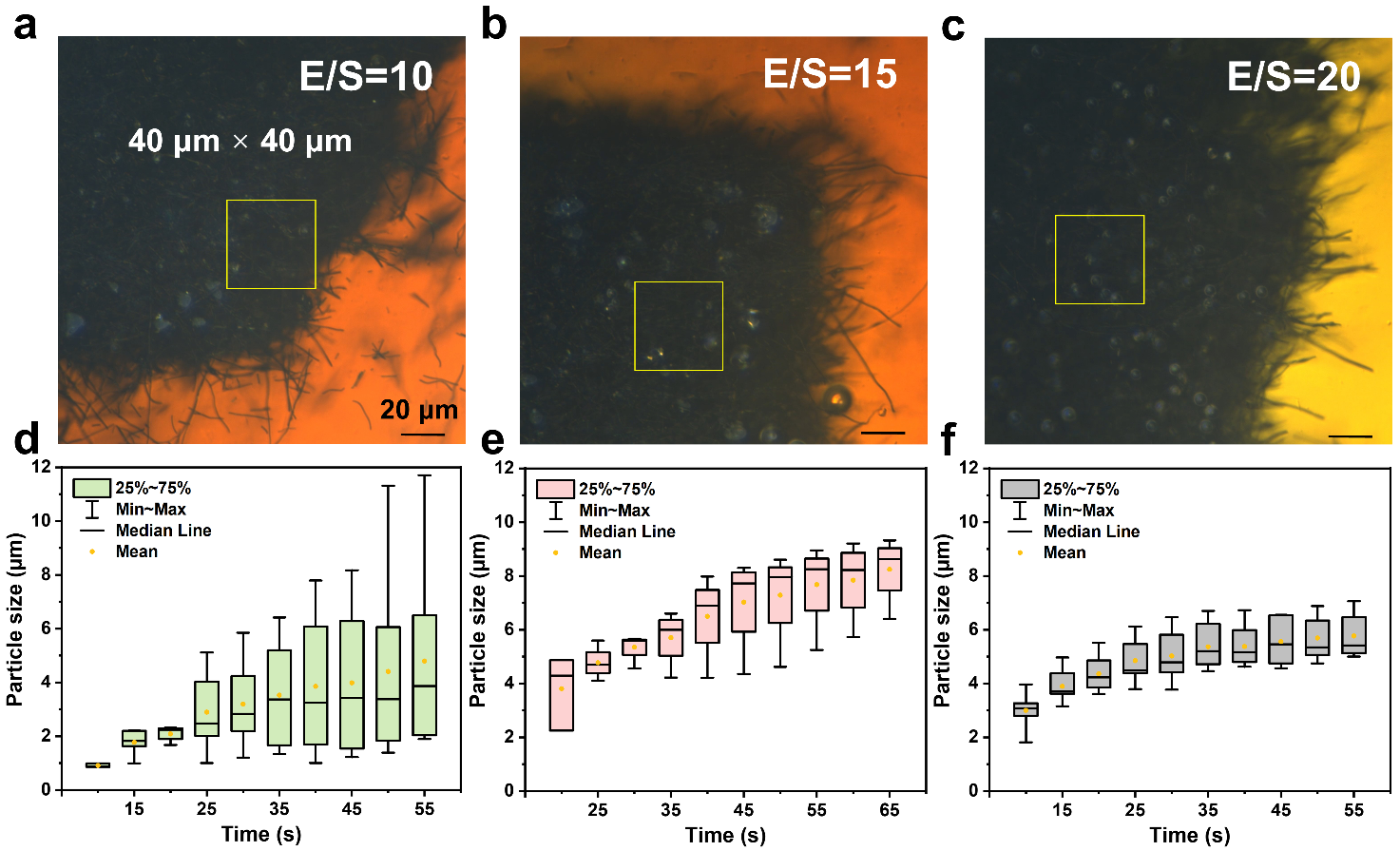


**Figure S5** (a-c) Area selection for calculation marked with yellow frames and (d-f) liquid sulfur particle size analysis with different E/S ratio of 10, 15 and 20 μL mg^-1^.


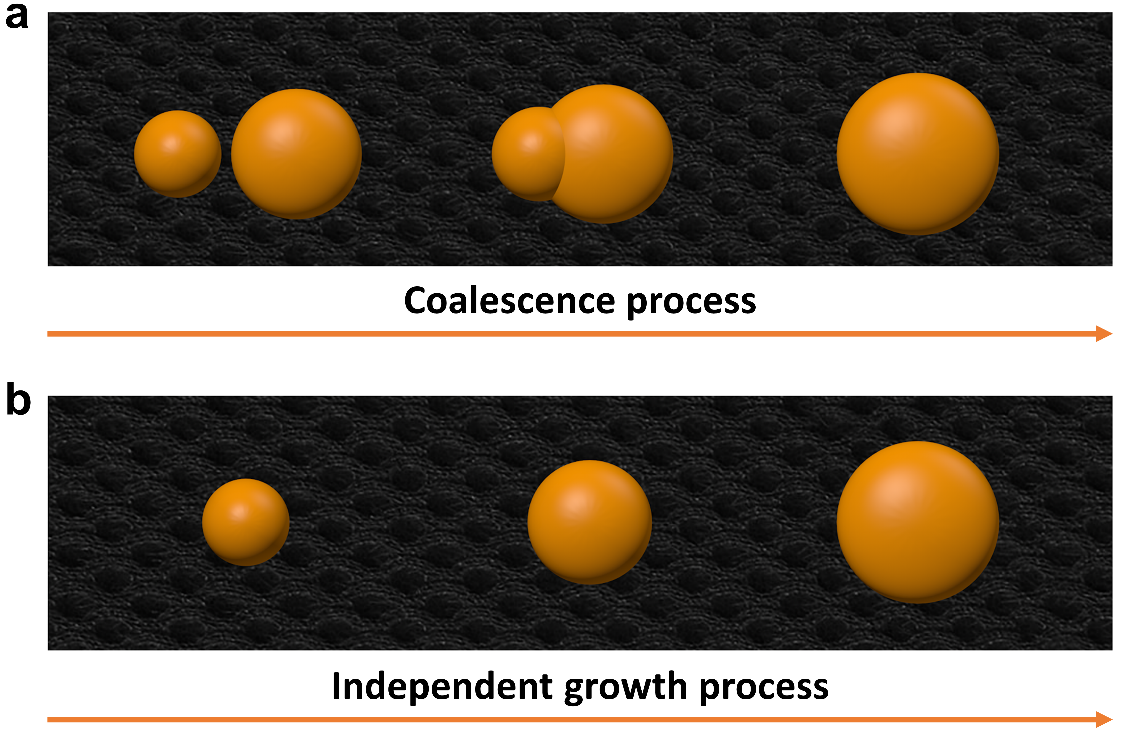


**Figure S6** Two kinds of mechanisms for liquid sulfur growing: (a) coalescence process, and (b) independent growth process.^[1]^


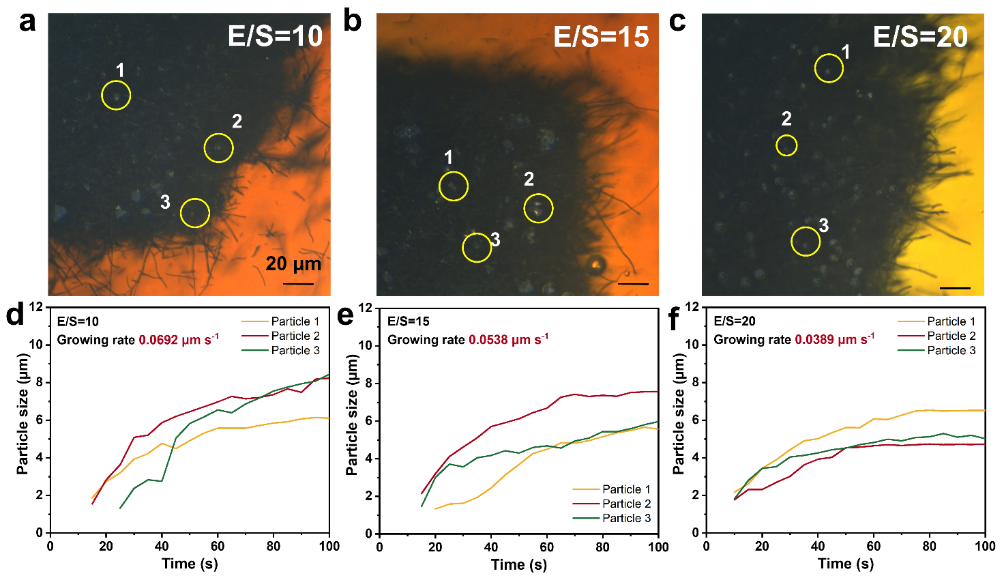


**Figure S7** (a-c) Three droplets chosen for each sample with E/S ratio of 10, 15 and 20 μL mg^-1^. (d-f) Liquid sulfur growing rate with E/S ratio of 10, 15 and 20 μL mg^-1^.


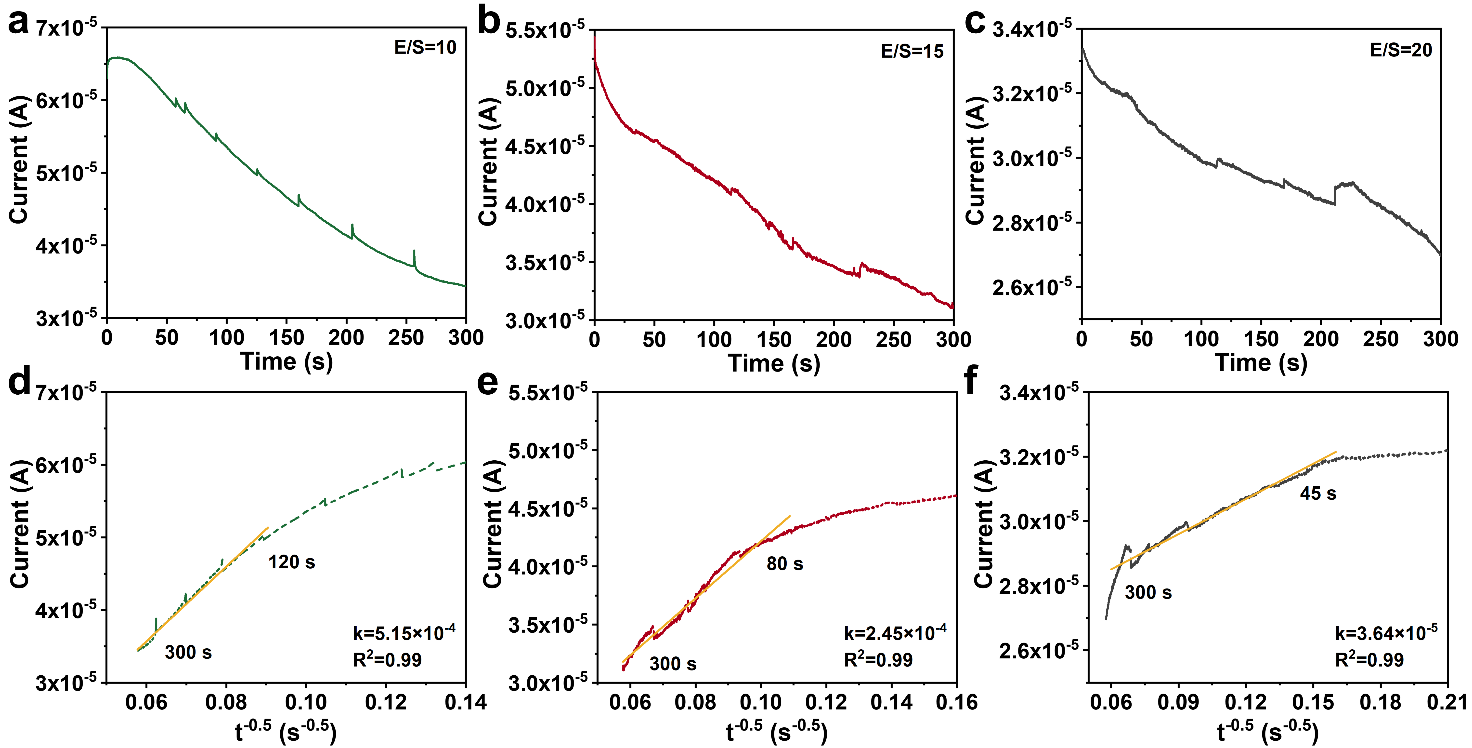


**Figure S8** (a-c) Reaction current and (d-f) linear fitting of the current to t^-0.5^ for the sample with E/S ratio of 10,15 and 20 μL mg^-1^.


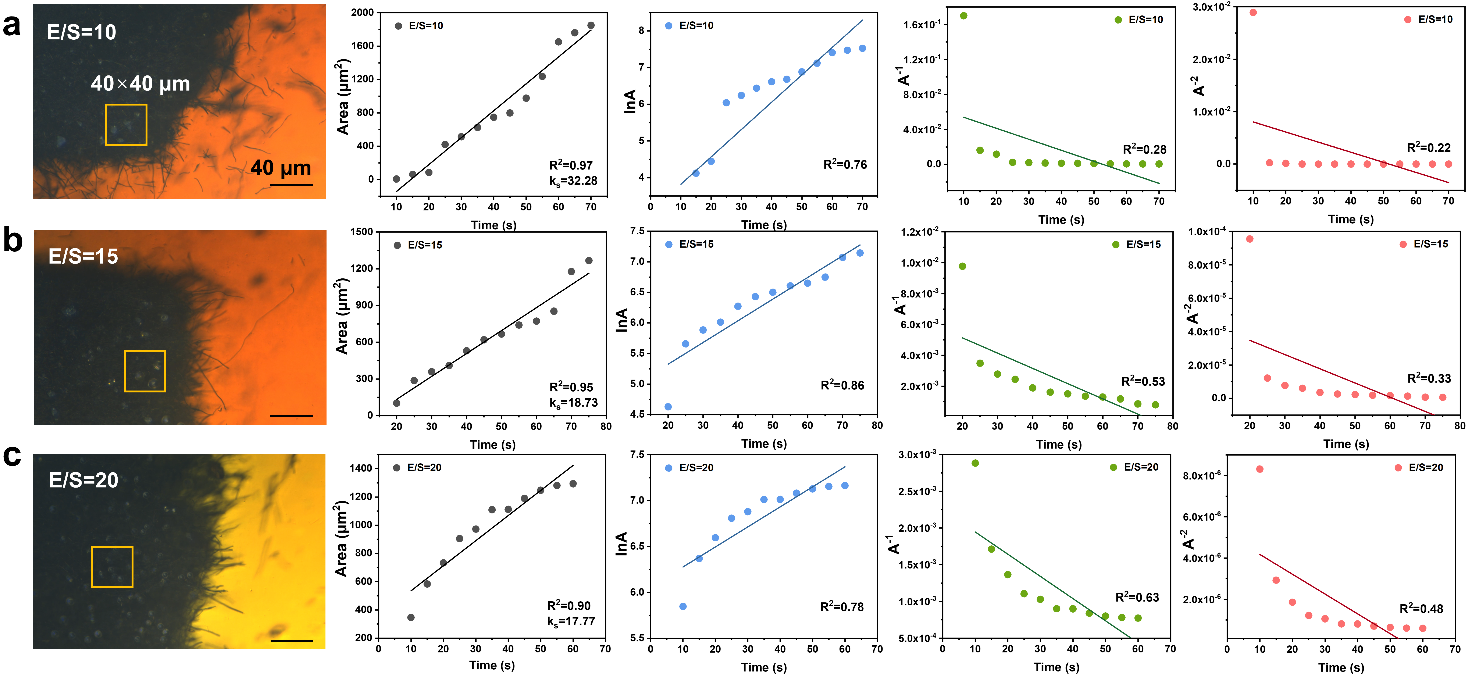


**Figure S9** Optical images of micro-cells and linear fitting curves of liquid sulfur area change with time: (a) E/S ratio=10 μL mg^-1^, (b) E/S ratio=15 μL mg^-1^, (c) E/S ratio=20 μL mg^-1^.


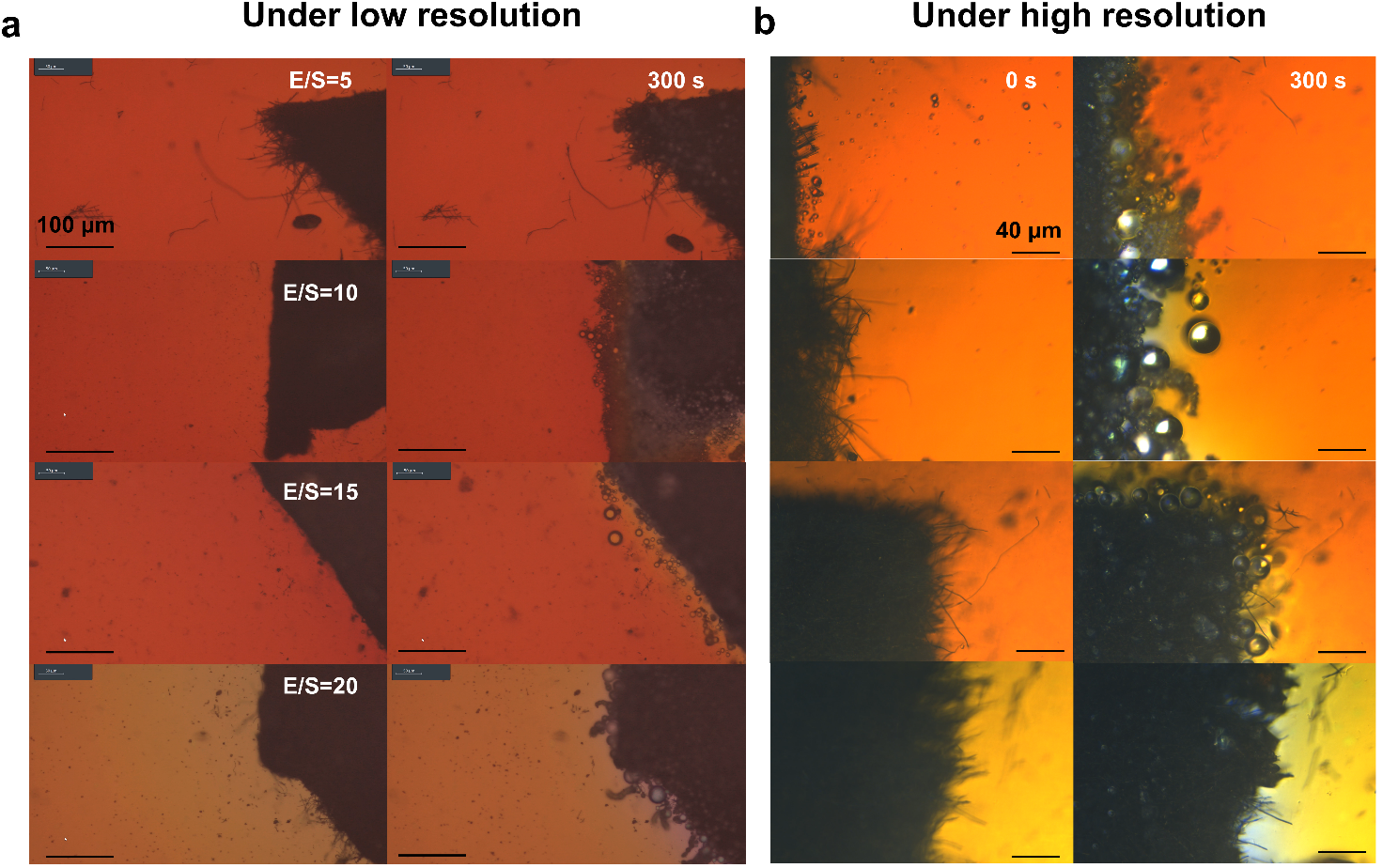


**Figure S10** (a) Optical images taken under low resolution (20× microscope objective) and (b) high resolution (50× microscope objective) with different E/S ratios.


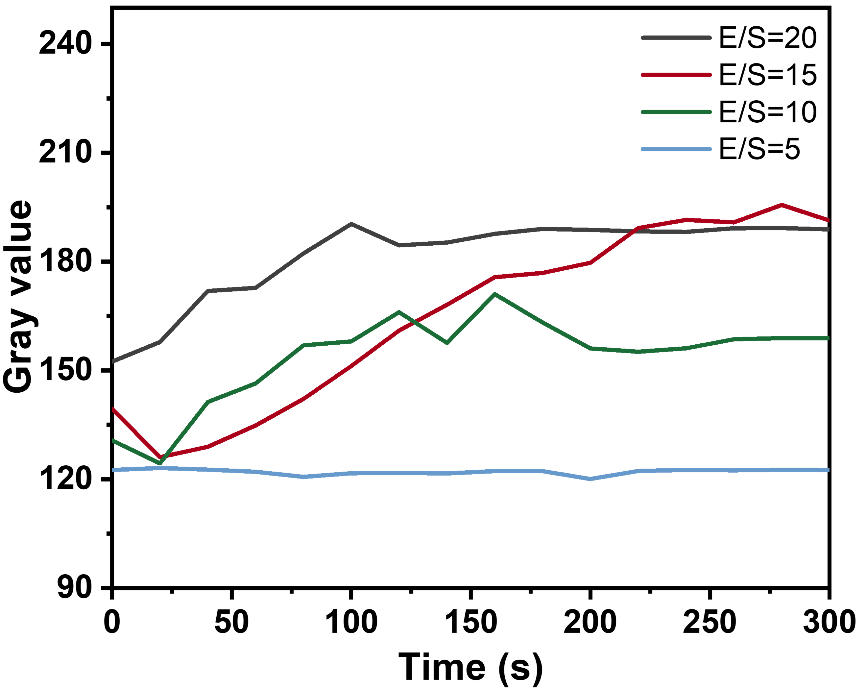


**Figure S11** Gray value changes of the white minimal selection along with charging time in Figure S2.


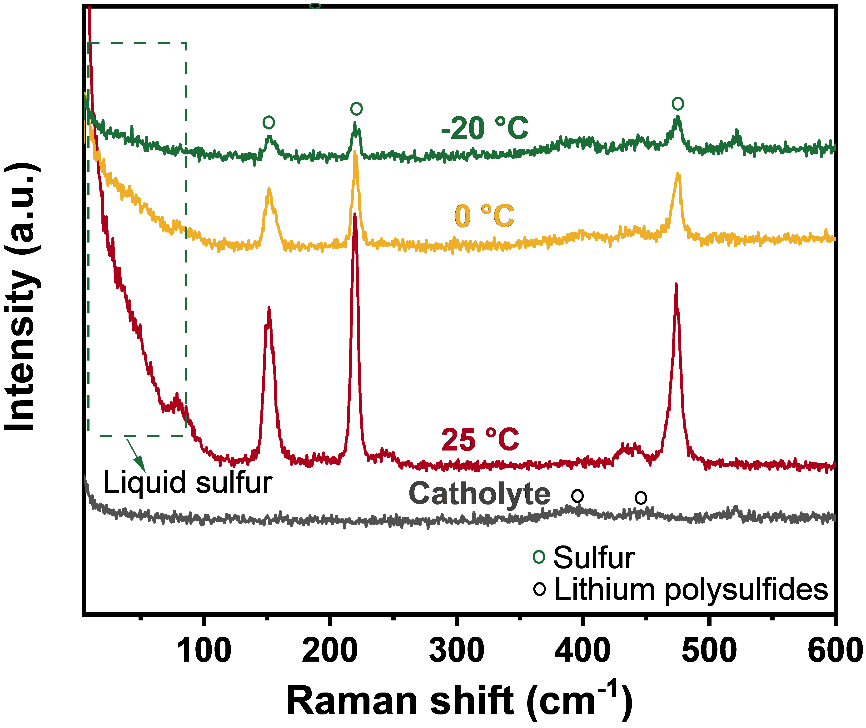


**Figure S12** Raman tests under different temperatures with E/S ratio of 5 μL mg^-1^.


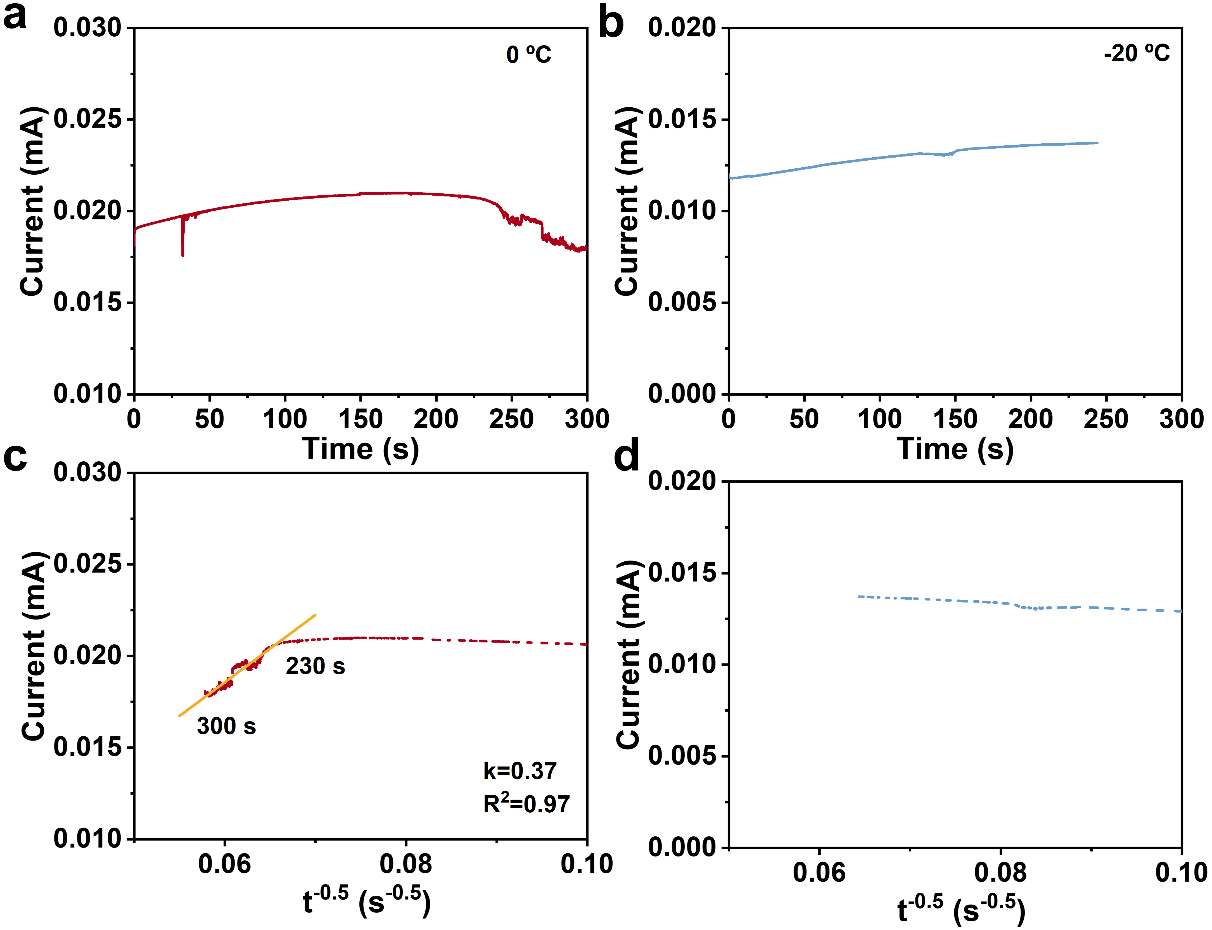


**Figure S13** (a, b) Reaction current and (c, d) linear fitting of the current to t^-0.5^ for the optical cells with E/S ratio of 5 μL mg^-1^ under 0 and -20 °C.


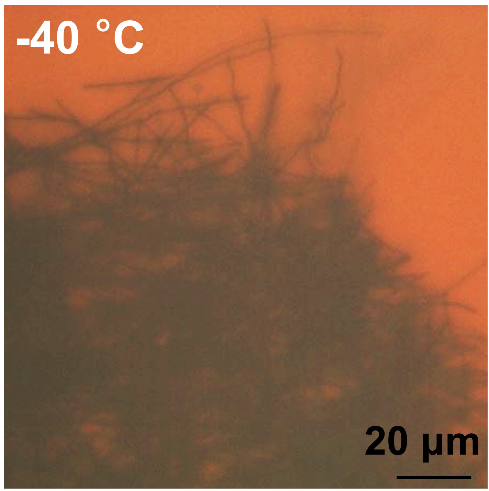


**Figure S14** Optical image of micro-cell with E/S ratio of 5 μL mg^-1^ under -40 ºC.


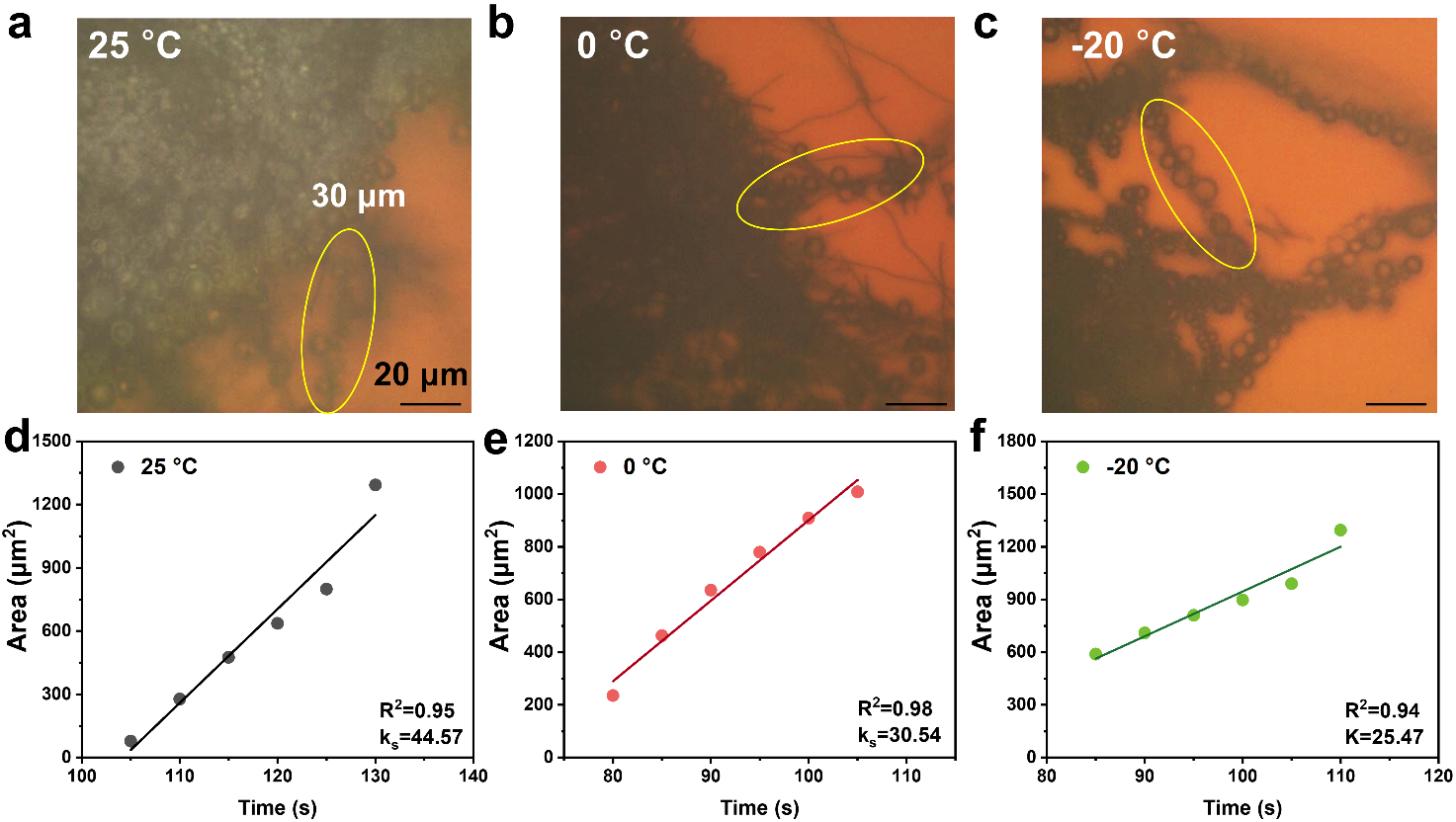


**Figure S15** (a-c) Optical images with E/S ratio of 5 μL mg^-1^ in room temperature, 0 and -20 ºC. The selected fibers are marked with yellow circles. (d-f) Reaction order and reaction constant calculation in room temperature, 0 ºC and -20 ºC.


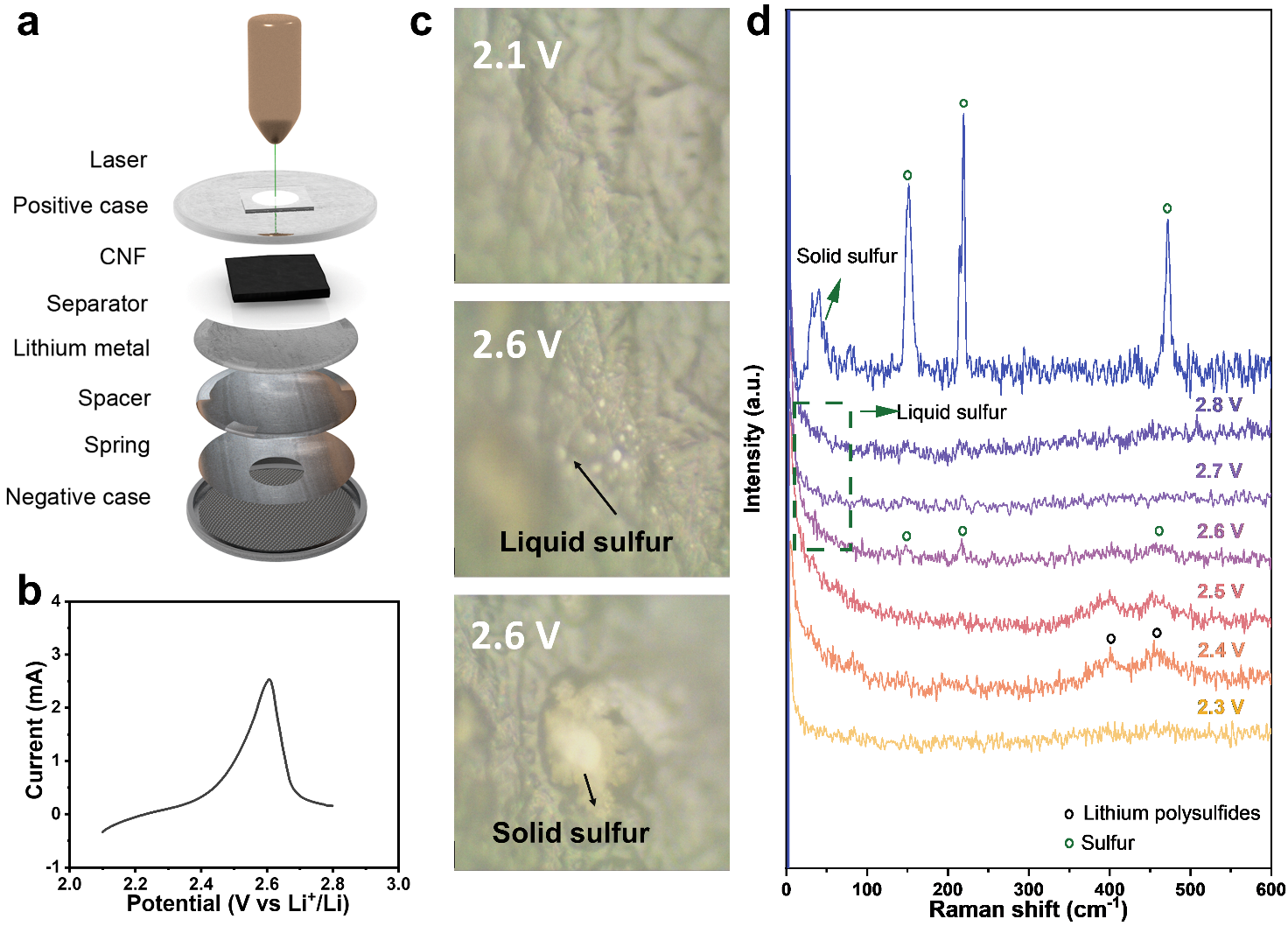


**Figure S16** (a) Scheme of coin cell tested through in situ Raman. (b) LSV curve of coin cell with E/S ratio of 10 μL mg^-1^, the scan rate is 1 mV s^-1^. (c) Optical image taken at 2.1 V and 2.6 V for liquid sulfur and solid sulfur. (d) In situ Raman curves at different charging depths, from 2.3 V to 2.8 V.


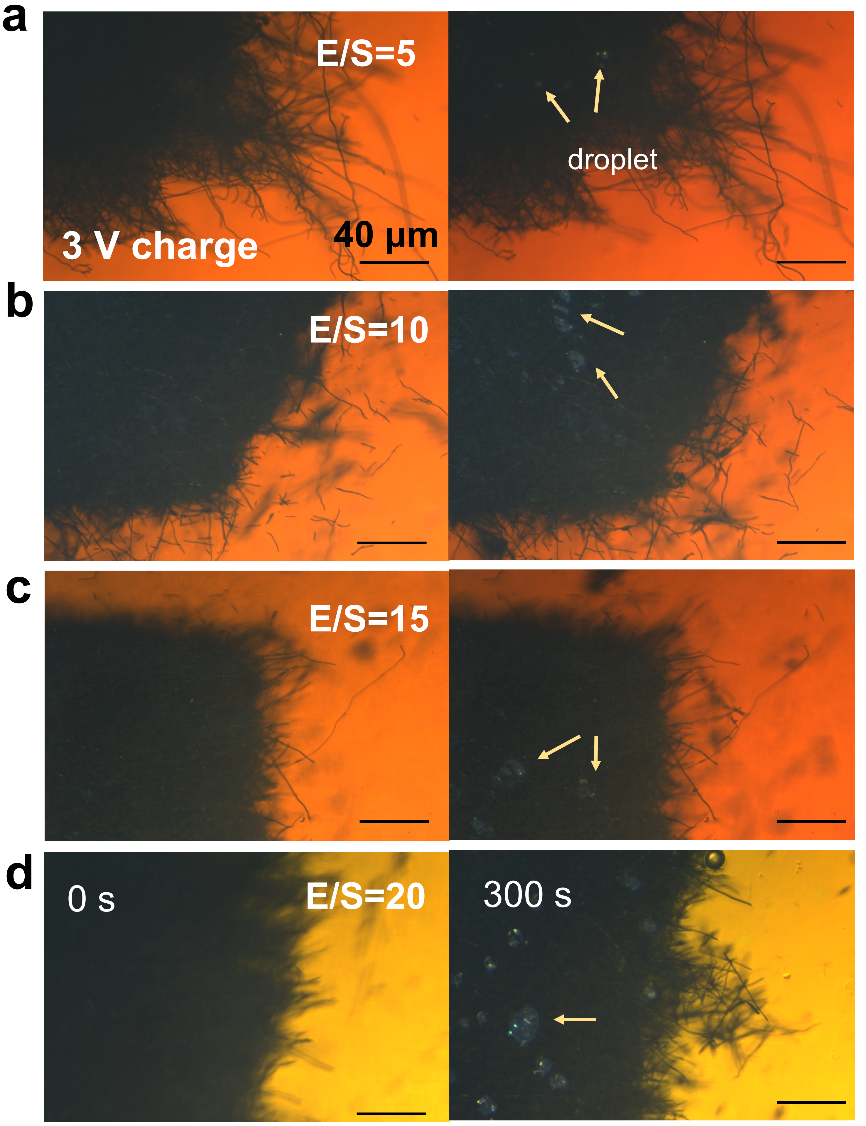


**Figure S17** Optical images taken for micro-cells with E/S ratios of 5, 10, 15 and 20 μL mg^-1^ with a constant voltage of 3 V. The liquid sulfur droplets are pointed out with yellow arrows.

**Table S1** Calculation of the liquid sulfur growing dynamics.

| **E/S ratio (μL mg^-1^)** | | **5** (15-60 s) | **10** (10-55 s) | **15** (20-65 s) | **20** (10-55 s) |
| --- | --- | --- | --- | --- | --- |
| **Initial size (μm)** | Mean | 0.907 | 0.912 | 3.805 | 3.002 |
|  | Median | 0.793 | 0.886 | 4.297 | 3.077 |
| **Size after 45 s’ charging (μm)** | Mean | 5.487 | 4.796 | 8.243 | 5.770 |
|  | Median | 5.257 | 3.862 | 8.627 | 5.423 |
| **Mean deviation (μm/μm)** | | 0.285/0.795 | 0.446/1.49 | 1.03/0.921 | 0.429/0.626 |
| **Standard deviation (%/%)** | | 37.4/97.6 | 56.2/191 | 137/127 | 64.6/76.3 |

**Table S2** Calculation of the independent sulfur growing kinetics.

| **E/S ratio (μL mg^-1^)** | | **Initial size (μm)** | **End size (μm)** | **Growing rate (μm s^-1^)** | **Average growing rate (μm s^-1^)** |
| --- | --- | --- | --- | --- | --- |
| **5** | Charge transfer (15-100 s) | 2.030 | 6.676 | 0.0547 | 0.0547 |
| **10** | Charge transfer (15-100 s) | 1.708 | 7.592 | 0.0692 | 0.0692 |
| **15** | Charge transfer (15-80 s) | 1.809 | 5.997 | 0.0644 | 0.0538 |
|  | Diffusion-controlled (80-100 s) | 5.997 | 6.378 | 0.0190 |  |
| **20** | Charge transfer (10-45 s) | 1.914 | 4.597 | 0.0766 | 0.0389 |
|  | Diffusion-controlled (45-100 s) | 4.597 | 5.419 | 0.0149 |  |

**Table S3** Calculation of diffusion coefficients D with every E/S ratio.

| **E/S ratios (μL mg^-1^)** | **5** | **10** | **15** | **20** |
| --- | --- | --- | --- | --- |
| **k (A s^-0.5^)** | 1.86×10^-4^ | 5.15×10^-4^ | 2.45×10^-4^ | 3.64×10^-5^ |
| **A (cm^2^)** | 0.0198 | 0.0220 | 0.0233 | 0.0171 |
| **C (mol cm^-3^)** | 7.8×10^-4^ | 4×10^-4^ | 2.6×10^-4^ | 2×10^-4^ |
| **D (cm^2^ s^-1^)** | 1.22×10^-8^ | 2.89×10^-7^ | 1.38×10^-7^ | 9.55×10^-9^ |

**Table S4** Analysis of Li_2_S_8_ concentration and the percent of concentration decay after 300 s’ reaction.

| **E/S ratio**  **(μL mg^-1^)** | **Beginning point (300 s)** | | | **Ending point (300 s)** | | |
| --- | --- | --- | --- | --- | --- | --- |
|  | Gray value | Concentration  (mol L^-1^) | Decay | Gray value | Concentration  (mol L^-1^) | Decay |
| **5** | 97.9 | ~0.78 | ~0% | 102.0 | ~0.78 | ~0% |
| **10** | 141.1 | 0.277 | 29.2% | 122.0 | 0.392 | 0.02% |
| **15** | 163.6 | 0.179 | 31.2% | 146.7 | 0.250 | 3.85% |
| **20** | 208.8 | 0.0253 | 87.3% | 175.7 | 0.134 | 33.0% |

**Supplementary note 1**: Calculation of diffusion coefficient D

Cottrell equation can be used to describe the change in current towards time when giving a certain potential on a planar electrode.^[2-3]^ The current depends on the rate that the reactant diffuses to the electrode. Thus, the reaction can be regarded as ‘diffusion-controlled’, which can be described as the following equation:

$i=\frac{nFAC_{j}^{0}\sqrt{D_{j}}}{\sqrt{\pi t}}$ (1)

Where *i* is the current (the unit is A), *n* is the number of electrons (n is 2 in this work for S_8_^2-^), *F* represents Faraday constant (96485 C mol^-1^), *A* represents the area of the planar electrode (the unit is cm^2^), *C* means the initial concentration of reactants (the unit is mol cm^-3^), *D* is diffusion coefficient (the unit is cm^2^ s^-1^) and t is the time (the unit is s). This equation can be simplified to equation (2):

$i=kt^{-\frac{1}{2}}$ (2)

If the reaction is diffusion-controlled, there should be a linear relation between the reaction current towards t^-0.5^. By mathematical fitting, k can be obtained for the calculation of the diffusion coefficient D.

**Supplementary note 2**: Calculation of the relation between the gray value and Li_2_S_8_ concentration

Grayscale images are used to measure the color depth in biology field, through which the hue and saturation information can be eliminated while the luminance of image is retained, which is beneficial for comparison.^[4]^ The color of Li_2_S_8_ catholyte changes with the increase of its concentration, so this method can be applied to figuring out the relation between them.

All RGB images in **Figure 3a** were taken with the same conditions of the microscope and were background subtracted to correct for uneven lighting in the images. Then they were converted to 8-bit images for grayscale analyzing. **Figure 3b** shows the fitting result: y=240.9x^2^-342.3x+217.3. There is a quadratic relation between the gray value and Li_2_S_8_ concentration, but the three coefficients are not certain that applies to all conditions, because the image color is influenced by several parameters of the microscope, such as light, exposure time, degree of blurry, etc. Therefore, the data in **Figure 3g-j** has been calibrated by this formula to eliminate the influence of these factors in this work.

**Supplementary Movies**

**Movie 1**: Sulfur generated on CNF with E/S ratio of 20. Video playback speed is 16 times faster than the original video.

**Movie 2**: Sulfur generated on CNF with E/S ratio of 15. Video playback speed is 16 times faster than the original video.

**Movie 3**: Sulfur generated on CNF with E/S ratio of 10. Video playback speed is 16 times faster than the original video.

**Movie 4**: Sulfur generated on CNF with E/S ratio of 5. Video playback speed is 16 times faster than the original video.

**Movie 5**: Sulfur generated on CNF with E/S ratio of 5 in room temperature. Video playback speed is 16 times faster than the original video.

**Movie 6**: Sulfur generated on CNF with E/S ratio of 5 in 0 ºC. Video playback speed is 16 times faster than the original video.

**Movie 7**: Sulfur generated on CNF with E/S ratio of 5 in -20 ºC. Video playback speed is 16 times faster than the original video.

**Reference**

[1] F. Shi, X. Guo, C. Chen, L. Zhuang, J. Yu, Q. Qi, Y. Zhu, Z.-L. Xu, S. P. Lau, *Nano Lett.* **2023**, *23*, 7906-7913.

[2] I. J. Suárez, T. F. Otero, M. Márquez, *J. Phys. Chem. B* **2005**, *109*, 1723-1729.

[3] M. Levi, E. Markevich, D. Aurbach, *Electrochim. Acta* **2005**, *51*, 98-110.

[4] S. Mas, A. Torro, N. Bec, L. Fernández, G. Erschov, C. Gongora, C. Larroque, P. Martineau, A. de Juan, S. Marco, *Anal. Chim. Acta* **2019**, *1074*, 69-79.
